# Computational modeling identifies key gene regulatory interactions underlying phenobarbital-mediated tumor promotion

**DOI:** 10.1093/nar/gkt1415

**Published:** 2014-01-23

**Authors:** Raphaëlle Luisier, Elif B. Unterberger, Jay I. Goodman, Michael Schwarz, Jonathan Moggs, Rémi Terranova, Erik van Nimwegen

**Affiliations:** ^1^Discovery and Investigative Safety, Novartis Institutes for Biomedical Research, 4057 Basel, Switzerland, ^2^Department of Toxicology, Institute of Experimental and Clinical Pharmacology and Toxicology, University of Tübingen, 72074 Tübingen, Germany, ^3^Department of Pharmacology and Toxicology, Michigan State University, MI 48824, USA and ^4^Biozentrum, University of Basel and Swiss Institute of Bioinformatics, 4056 Basel, Switzerland

## Abstract

Gene regulatory interactions underlying the early stages of non-genotoxic carcinogenesis are poorly understood. Here, we have identified key candidate regulators of phenobarbital (PB)-mediated mouse liver tumorigenesis, a well-characterized model of non-genotoxic carcinogenesis, by applying a new computational modeling approach to a comprehensive collection of *in vivo* gene expression studies. We have combined our previously developed motif activity response analysis (MARA), which models gene expression patterns in terms of computationally predicted transcription factor binding sites with singular value decomposition (SVD) of the inferred motif activities, to disentangle the roles that different transcriptional regulators play in specific biological pathways of tumor promotion. Furthermore, transgenic mouse models enabled us to identify which of these regulatory activities was downstream of constitutive androstane receptor and β-catenin signaling, both crucial components of PB-mediated liver tumorigenesis. We propose novel roles for E2F and ZFP161 in PB-mediated hepatocyte proliferation and suggest that PB-mediated suppression of ESR1 activity contributes to the development of a tumor-prone environment. Our study shows that combining MARA with SVD allows for automated identification of independent transcription regulatory programs within a complex *in vivo* tissue environment and provides novel mechanistic insights into PB-mediated hepatocarcinogenesis.

## INTRODUCTION

Aberrant activity of transcription factors (TFs) is a hallmark of both human (1–3) and mouse (4) hepatocarcinogenesis and is considered as a key intrinsic regulatory mechanism underlying epigenetic reprograming associated with cancer development (5). Non-genotoxic carcinogens (NGC) are a group of compounds that do not directly affect DNA (6), but that produce perturbations in the gene expression and epigenetic state of cells (7–9) which, if given in sufficient concentration and duration, facilitate tumor formation, typically through the promotion of pre-existing neoplastic cells into neoplasms (10,11). However, little is known about the regulatory mechanisms that underly the tumor promotion by NGC, particularly regarding the early regulatory changes in response to the carcinogen.

The anticonvulsant phenobarbital (PB) is a well-established rodent NGC that has been extensively used to investigate the promotion of liver tumors (12–14). PB accomplishes its diverse effects on liver function, at least in part, by promoting nuclear translocation of the constitutive androstane receptor (CAR) (15) through inhibition of Epidermal Growth Factor Receptor (EGFR) signaling (16). CAR activation is required for the acute and the chronic response to PB treatment and for liver tumor formation elicited upon prolonged PB treatment (17–21). In addition to this crucial role of CAR, when liver tumors are promoted through PB treatment in combination with an initial treatment with diethylnitrosamine (DEN), >80% of the resulting tumors harbor activating mutations in β-catenin (22) that stabilize β-catenin, leading to enhanced nuclear translocation and subsequent target gene activation (23–27).

Apart from the crucial roles for CAR and β-catenin in PB-mediated liver tumor promotion, little is known about additional transcriptional regulators that orchestrate the complex and dynamic PB-mediated gene expression programs associated with early molecular responses to PB treatment (28) and long-term PB tumorigenic effects (12–14).

In this study, we have elucidated gene regulatory interactions underlying dynamic PB-mediated transcriptional responses during the early stages of liver non-genotoxic carcinogenesis by integrating multiple gene expression datasets from independent *in vivo* mouse PB studies. Our primary dataset consists of an early kinetic study (seven time points across 91 days of PB treatment) originally designed to investigate the temporal sequence of molecular and histopathological perturbations during the early stages of PB-mediated liver tumor promotion *in vivo* (9, 28). Several challenges are associated with the extraction of key gene regulatory interactions from gene expression time course data. First, we needed to identify the relative contributions (activities) of specific transcriptional regulators underlying the observed genome-wide gene expression changes. This was achieved using our recently developed motif activity response analysis [MARA, (29)]. MARA capitalizes on sophisticated computational methods, developed over the last decade (30), that allow comprehensive prediction of binding sites for hundreds of mammalian TFs across all mammalian promoters (31). Using such computational predictions, MARA models observed gene expression patterns explicitly in terms of the predicted regulatory sites and uses this to infer the regulatory activities of TFs. A number of recent studies (32–43) demonstrate that this approach can successfully identify key regulators *ab initio* across different model systems of interest.

A second challenge was to disentangle the complex range of PB-mediated gene expression programs in mouse liver tissue that are associated with distinct biological events including xenobiotic responses, tumor promotion and tumorigenesis.

Here, we show that combining MARA with singular value decomposition (SVD) allows for automated disentangling of independent transcription regulatory programs within a complex *in vivo* tissue environment. We were able to successfully infer key gene regulatory proteins for xenobiotic responses, tumor promotion and end-stage tumors as well as assess their genetic dependence on CAR and β-catenin signaling pathways.

Collectively, our analyses provide novel mechanistic insights into PB-mediated tumor promotion in the mouse liver, including a proposed role of E2F and ZFP161 in regulating PB-mediated hepatocyte proliferation at both early and tumor stages and progressive PB-mediated suppression of ESR1 activity that likely contributes to the development of a tumor-prone environment.

## MATERIALS AND METHODS

### Gene expression datasets and Affymetrix GeneChip processing

**Figure 1.**
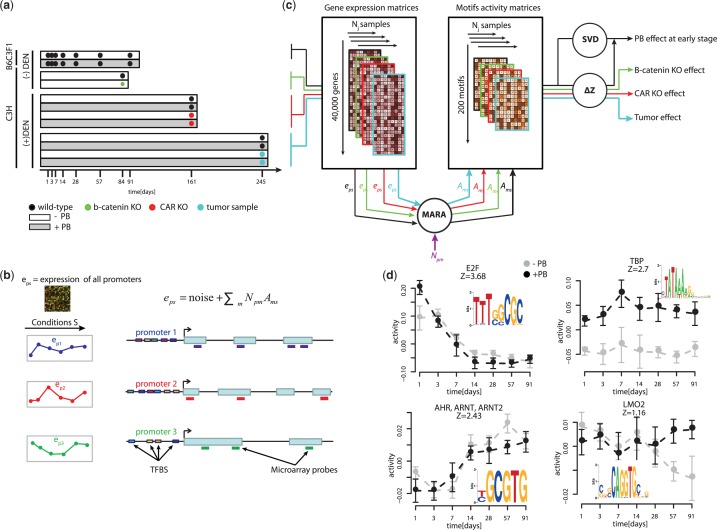
Overview of the analysis strategy for predicting TF activities from gene expression data. (**a**) Schematic representation of the four toxicogenomic datasets used in this study. Each row corresponds to an independent experimental treatment with gray indicating PB treatment and white indicating control treatment. Time is indicated along the horizontal axis on the bottom and the black dots indicate the times at which samples were taken for transcriptomic profiling. Colored dots indicate whether the study involved WT animals (black), liver-conditional β-catenin null mice (green), CAR null mice (red) or tumor cells (blue). The mouse strain used in each of the studies as well as the presence or absence of DEN treatment is indicated on the left-hand side. Multiple biological replicates were performed for each of the studies. (**b**) Outline of the MARA approach. Using measured intensities of micro-array probes together with known gene structures, MARA first estimates the log-expression *e_ps_* from each promoter *p* in each sample *s*. Second, MARA makes uses of comprehensive computational predictions of transcription binding sites in mammalian promoters, with *N_pm_* denoting the total number of predicted regulatory sites for motif *m* in promoter *p*. MARA then models the expression levels *e_ps_* as a linear function of the numbers of computationally predicted binding sites *N_pm_* and unknown ‘motif activities’ *A_ms_* of each motif *m* in each sample *s*. That is, the algorithm uses the linear model to infer the motif activities *A_ms_*. (**c**) Overview of the computational analysis applied to our combination of datasets. First, MARA is applied to all expression datasets and motif activities *A_ms_* are inferred for all motifs across all samples. These motif activities are then further subjected to SVD analysis and analysis of various contrasts (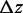). (**d**) Examples of inferred motif activities along the time 91-day time course. Each panel corresponds to one motif, with the name of the TF indicated on top of the panel and its sequence logo as an inset. Black and gray lines are predicted activities in PB-treated and control samples, respectively.

A library of 109 genome-wide messenger RNA (mRNA) expression patterns was compiled from four different studies (Figure 1a). In all four studies gene expression was profiled using Affymetrix GeneChip MOE-4302 (Affymetrix, Santa Clara, CA, USA). The analysis of the micro-array data was done with the R statistical package, version 2.13 (2005) and Bioconductor libraries, version 1.4.7 (44).

### From gene expression matrices to motif activity matrices

Matrices of activities for 189 mammalian regulatory motifs across all samples were inferred from the RMA-normalized expression matrices using the MARA algorithm (29) (Figure 1b and c). MARA models genome-wide gene expression patterns in terms of predicted functional Transcription Factor Binding Sites (TFBSs) within proximal promoter regions (running from −300 to +100 relative to transcription start) of the 40 300 promoters. The model assumes that the expression *e_ps_* of a promoter *p* in sample *s* is a linear function of the predicted numbers of binding sites *N_pm_* for each motif *m* in promoter *p* and the (unknown) activities *A_ms_* of each of the motifs *m* in sample *s*, i.e.

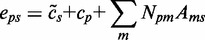

where *c_p_* reflects the basal activity of promoter *p* and 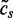 is a normalization constant corresponding to the total expression in sample *s*. The activities *A_ms_*, as well as error bars 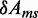 on these activities, are thus inferred from the measured expression data *e_ps_* and the predicted binding sites *N_pm_*. The number of functional TFBSs *N_pm_* was predicted using the Bayesian regulatory site prediction algorithm MotEvo, which incorporates information from orthologous sequences in six other mammals and uses explicit models for the evolution of regulatory sites (30). The 189 regulatory motifs represent binding specificities of roughly 350 different mouse TFs. Besides the motif activities, MARA also calculates a *z*-score quantifying the significance of each motif in explaining the observed expression variation across the samples, the target genes of each motif, and the sites on the genome through which the regulators act on their targets.

Formally, the activity *A_ms_* corresponds to the amount by which the expression *e_ps_* would be reduced if a binding site for motif *m* in promoter *p* were to be removed. Thus, an increasing activity is inferred when its targets show on average an increase in expression, that cannot be explained by the presence of other motifs in their promoters. The details of the method are described elsewhere (29). An overview of the analysis strategy and an outline of the MARA approach are depicted in Figure 1.

### Detection of differential motif activity between pairs of conditions

We quantified the differential motif activity between two conditions using a *z*-statistic as

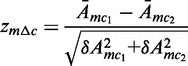

where 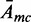 is the averaged motif activity profile over replicates for condition *c* and motif *m* and 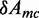 is the standard error on the corresponding motif activity, which is computed using a rigorous Bayesian procedure (Balwierz, PJ. et al, manuscript under review). The *z*-values quantify the evidence for a change in regulatory activity of the motif between the two conditions. That is, if 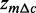 is highly positive it indicates that predicted targets of motif *m* are upregulated in condition *c*_1_ relative to condition *c*_2_, in a way that cannot be explained by the activities of other regulators. We consider motifs differentially active if 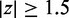.

**Figure 2.**
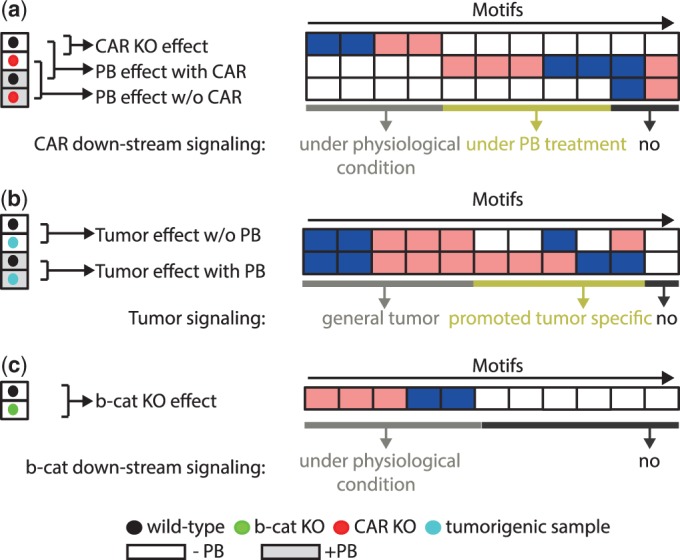
Schematic representation of contrasts applied for differential motif activity analysis of each dataset. The color of the dot indicates whether the sample is WT (black), β-catenin KO (green), CAR KO (red) or tumor (blue). White boxes correspond to control samples and gray boxes to PB-treated samples. The arrows show which pairs of samples are compared for each contrast and point to corresponding rows with example motif activity changes (blue corresponding to downregulation 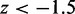, pink to upregulation 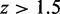 and white no significant change 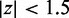). (**a**) Motif activities from the CAR KO study are compared to identify regulators downstream of the CAR pathway under physiological conditions and under PB treatment. (**b**) Motif activities from the tumor study are compared to identify promoted tumor-specific regulators. (**c**) Motif activities from the β-catenin KO study are compared to identify downstream regulators of the β-catenin pathway under physiological conditions.

In order to avoid any confounding batch effects, we only calculate differential activities across conditions from the same dataset. Comparing activities between treated and control samples at different timepoints of the kinetic study allowed for the identification of PB-mediated dysregulated TFs at the early stage of PB treatment. Comparison of activities between wild-type (WT) and CAR/β-catenin null in physiological conditions, i.e. without treatment, identified motifs whose activities are modulated upon KO of the respective TF (Figure 2a and c). We consider such motifs to be downstream of the β-catenin/CAR pathways in physiological conditions. Similarly, comparison of activities between PB-treated and non-treated samples allowed for the identification of motifs that are dysregulated by PB treatment. By further comparing the changes in motif activities upon PB treatment for both WT and CAR-null samples, we can identify motifs dysregulated by PB in a manner that is independent of CAR signaling and motifs whose dysregulation is downstream of CAR (Figure 2a and c). Comparison of activities between promoted tumors and surrounding PB-treated tissue identified motifs dysregulated in promoted tumors; comparison of activities between non-promoted tumors and surrounding non-treated tissue identified motifs dysregulated in liver tumors irrespective of PB treatment. Motifs uniquely dysregulated in promoted tumors were classified as promoted tumor-specific regulators (Figure 2b).

### Characterization of PB-mediated early motif activity profiles

#### SVD of the motif activities

We performed SVD of the activities of the 189 motifs across the seven timepoints in PB- and vehicle-treated livers, i.e. a matrix *A* containing 189 rows and 14 columns. SVD resulted in a decomposition, 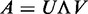, where Λ is a diagonal matrix containing the singular values, *U* and *V* contain the orthonormal bases defined by right and left singular vectors of *A*, respectively. Each motif activity profile 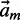 with 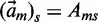 can be thought of as a linear combination of the right singular vectors 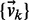.

#### Visualization and interpretation of the SVD results

To visualize the right singular vectors 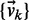, we plotted the activities *v_ks_* on the vertical axis as a function of the time corresponding to each sample *s* on the horizontal axis and coloring all samples corresponding to PB treatment black, and those corresponding to control-treatment gray, e.g. Figure 1d. This visualization facilitated the biological interpretation of the singular vectors. Biological interpretation was further facilitated by identification of the regulatory motifs whose activity profiles correlate most strongly (either positively or negatively) with the activity profile of the singular vector.

### Identification of representative motifs of the singular vectors

As the right singular vectors form an orthonormal basis of the space of activity profiles, the projection of a given motif activity profile onto a right singular vector indicates how strongly the motif’s activity profile overlaps with the basis vector specified by the singular vector. The projections of the motif activity profiles 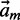 onto right singular vectors 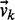 are calculated as 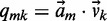 and these values are readily obtained from the SVD results as 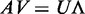 such that 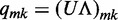.

We additionally computed Pearson correlations between the motif activity profiles 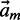 and the right singular vectors 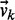. As the vectors 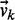 are linear combinations of the motif activity profiles 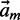 that are mean centered, i.e. 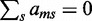, these are also mean centered. Consequently, the Pearson correlation coefficients can also be readily obtained from the SVD results as 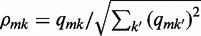. As the activity profiles of different motifs have different overall ‘lengths’, the projections and Pearson correlations do not carry identical information. Motifs with large activities tend to have high absolute projections with a given singular vector, even if the motif activity profile is not similar to the activity profile of the singular vector. In contrast, a motif with small activities will tend to have low projections, but may have a high correlation with a given singular vector.

In order to identify representative motifs for each singular vector, motifs were ranked according to both projection and correlation scores. The highest (most positive scores in both projection and correlation) and lowest (most negative scores in both correlation and projection) motifs were selected for each singular vector. As some degree of redundancy is present among regulatory motifs, we further refined our motifs selection in a systematic manner following criteria that are detailed in the ‘Results’ section.

#### Gene Ontology enrichment analysis

The DAVID Bioinformatics Resource (Database for Annotation, Visualization and Integrated Discovery) (45,46), version 6.7, sponsored by the National Institute of Allergy and Infectious Diseases (NIAID), NIH, was used to investigate the statistical enrichment of biological terms and processes associated with the predicted target genes of each motif of interest. We directly imported official gene symbols into DAVID, exported enrichment from biological pathways from Gene Ontology and Kyoto Encyclopedia of Genes and Genomes (KEGG), filtered out redundant terms and selected biological processes with *P*-value of enrichment <0.05.

## RESULTS

### Overview of liver toxicogenomic data from phenobarbital-treated mouse models

In order to investigate gene regulatory networks underlying early PB-mediated liver tumor promotion, we used four transcriptomic datasets which are illustrated in Figure 1a. Our primary dataset is composed of transcriptome profiling data from a PB kinetic study in B6C3F1 (livers from vehicle, i.e. control and PB-treated male mice at +1, +3, +7, +14, +28, +57 and +91 days of dosing). This dataset enabled us to investigate gene expression dynamics during the first 3 months of PB treatment. A second CAR knock-out (KO) study composed of transcriptome profiling data from livers of vehicle- and PB-treated C3H male WT and CAR-null mice (at +161 days of dosing) enabled us to investigate which of the responses to PB treatment were CAR-dependent at this later time point. A third tumor study consisting of samples from promoted (at +35 weeks of PB treatment) and non-promoted tumors as well as their related surrounding tissue from C3H male mice, enabled us to identify gene regulatory changes that were specific to promoted tumors, as opposed to being a shared feature of tumor tissues in general. Finally, a β-catenin KO study composed of livers from WT and β-catenin-null C3H male mice enabled us to investigate which of the identified TFs were downstream of β-catenin in physiological conditions. In both the CAR KO and tumor studies, mice were DEN-initiated at 4 weeks of age.

### Identifying PB-modulated activities of transcriptional regulators using MARA

MARA is a general method for inferring the activities of a large collection of mammalian TFs (as represented by their DNA binding ‘motifs’) by modeling gene expression data in terms of computationally predicted regulatory sites in promoters. The basic approach is illustrated in Figure 1b. Note that motif activities are inferred from the behavior of the expression levels, typically hundreds, of predicted ‘targets’ of the motif and do not directly involve analysis of the expression levels of the regulators themselves. This is especially useful in systems where TF activities are modulated through subcellular localization and post-translational modifications, rather than at the transcriptional level, e.g. such as the PB-mediated CAR nuclear translocation and induction of downstream transcriptional responses that we study here. Importantly, apart from inferring the motif activities *A_ms_*, MARA also rigorously infers error bars on these motif activities 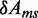, which allow to quantify to what extent motif activities are significantly varying across the samples for each motif. The overall significance of each motif *m* is then represented by a *z*-statistic (‘Materials and Methods’ section).

### TFs underlying early PB-mediated liver transcriptional dynamics

Figure 1d shows the activities of four motifs observed within the time course of control and PB-treated mice, illustrating the range of different profiles that can be observed. For example, the motif bound by the family of E2F TFs and the motif bound by AHR, ARNT and ARNT2 TFs both showed substantial changes in activity across the time course that are largely the same in the control and PB-treated animals, except for E2F’s activity at the first timepoint. In contrast, the TATA-box motif bound by TATA Binding Protein (TBP) exhibited almost constant activity across time but showed a strong shift in behavior between control and PB-treated animals. The LMO2 motif showed no significant activity for the first month of the time course but at later time points (during the last 2 months) there was a marked divergence between PB-treated and control animals.

#### SVD identifies four characteristic motif activity profiles underlying early PB-mediated transcriptional changes

Although it is possible to formulate biological interpretations and hypotheses for observed motif activity profiles on a case-by-case basis, it is unclear how this could be performed in a systematic and unbiased manner across a large number of motifs. This is especially challenging, because prior biological knowledge indicates that multiple biological processes, including completion of postnatal liver development, acute and sustained xenobiotic responses to PB treatment and tumor promotion, are occurring in parallel in our system. To address this problem, we applied a SVD approach to decompose the matrix of inferred motif activities *A_ms_* from the early kinetic study into linearly independent motif activity profiles that capture most of the variation in all motif activities.

**Figure 3.**
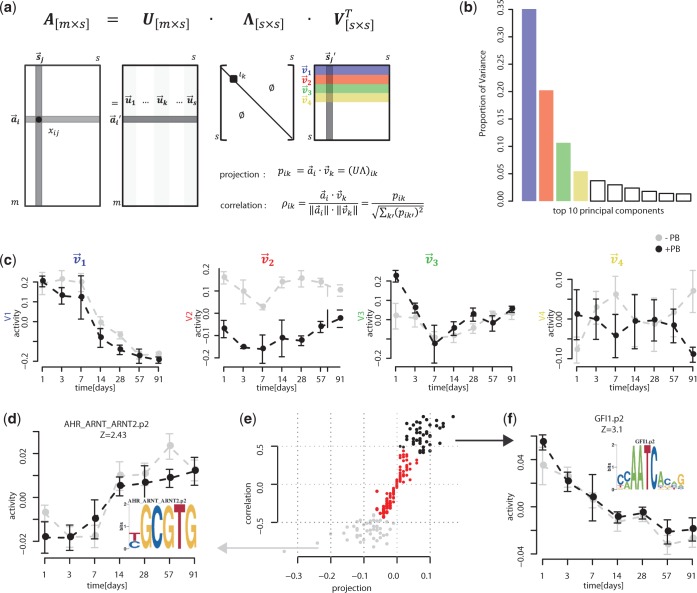
Overview of the analysis strategy for identifying key regulatory activities of the early PB-mediated transcriptional dynamics. (**a**) SVD factorizes the activity matrix of the early kinetic study: 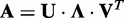, with the right singular vectors 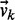 giving orthonormal motif activity profiles that capture most of the variation in activity profiles across all motifs. (**b**) Proportion of the variance of the motif activity matrix explained by the 10 first components. The first (blue bar), second (red bar), third (green bar) and fourth (yellow bar) components account for 35, 20, 10 and 5%, respectively of the variance. (**c**) Activity profiles of the first four right singular vectors 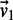 through 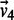. Gray points indicate activities for the control samples and black points indicate activities for the PB-treated samples. (**d, f**) Examples of motif activity profiles that contribute and correlate negatively and positively, respectively, with the first right singular vector (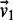). For each motif, a sequence logo representing its binding specificity is shown as an inset. (**e**) Scatter plot of the correlations 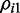 and projections 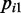 of all motifs *i* with the first right singular vector 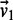. Gray and black dots depict negatively and positively selected motifs.

Over 70% of the variance in the activity matrix was explained by the first four components of the SVD as evidenced by the spectrum of singular values (Figure 3b). The activity profiles of the first four right singular vectors, 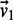 through 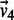, are shown in Figure 3c. The first right singular vector accounted for 35% of the variance and was characterized by an approximately constant positive activity early in the time course that decreased dramatically after 2 weeks. The activity profile of this first singular vector was identical in the PB-treated and control groups. The steep drop in activity after 2 weeks coincided with the completion of postnatal liver development in this study, as indicated by the transcriptional profile of the hepatoblast marker α-fetoprotein (*Afp*) (47,48) (Supplementary Figure S4). We thus propose that this characteristic motif activity profile is associated with postnatal liver development. This conclusion is supported by some of the motifs associated with this singular vector (see Supplementary Data). As this process is presumably not relevant for the process of non-genotoxic tumor promotion, we have not further focused on this singular vector and a characterization of its associated regulators is presented in the Supplementary Data.

The second singular vector accounted for 20% of the variance and was characterized by an activity profile that is almost entirely constant with time, but that showed a large difference between the PB-treated and vehicle-treated samples. This singular vector thus corresponds to a sustained xenobiotic response.

The third singular vector accounted for 10% of the variance and was characterized by a difference in activity between the control and treated group at Day 1 only; whereas activity in the control samples remained approximately constant in the first 3 days, activity was much higher at Day 1 and dropped significantly in PB-treated samples over the same initial phase. Given that PB mediates a transient mitotic response at Day 1 [also previously identified in other studies (18, 28)], we conclude that the biological pathway corresponding to this characteristic activity profile is the transient PB-mediated proliferative response.

Finally, the fourth singular vector accounted for 5% of the variance and was characterized by a divergence in the activity of the PB-treated and control groups in the last month of the 13-week time course. Given that this is the most significant singular value for differences between the PB-treated and control samples toward the end of the time course, we infer that this characteristic adaptive xenobiotic response activity profile might be an important contributor to the progressive creation of a tumor-prone environment.

In summary, we have shown that the behavior of regulatory motifs in the early stages of PB treatment are dominated by four characteristic activity profiles that account for >70% of variance of the motif activities and which correspond to the following fundamental biological processes: (i) the completion of postnatal liver development, (ii) a constant xenobiotic response, (iii) a PB-mediated acute mitogenic response and (iv) an adaptive xenobiotic response (late response to PB treatment).

#### Identification of representative motifs underlying the early dysregulated biological pathways

To determine motifs underlying the four characteristic motif activity profiles identified in the previous section, we selected motifs which contributed and correlated the most with each of the four singular vectors (Figure 3c, d, e and f). In this way we obtained, for each of the four singular vectors, two clusters of motifs with similar activity profiles, i.e. one correlating negatively with the singular vector and one correlating positively (Figure 3d and f). The advantage of extracting clusters of the most important regulatory motifs in this way, rather than simply clustering the motif activity profiles directly, is that many of the motif activity profiles contain components associated with different biological processes that are operating in parallel in our system. By first using SVD to identify the most significant characteristic activity profiles that are mutually ‘independent’, i.e. the singular vectors, we disentangle the regulatory activities associated with these different processes and cluster the motifs by the biological process.

We further refined the selection of the motifs associated with each singular vector as follows: (i) removing motifs for which the overall significance was too low (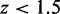 for motifs regulating postnatal liver development or the constant xenobiotic response and under 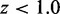 for motifs regulating the transient mitogenic response or the adaptive xenobiotic response); (ii) removing motifs whose cognate TFs were not expressed in the liver (log expression <6.0); (iii) using *z*-scores for the differential activity per time point between PB-treated and control samples, we required 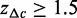 at minimum four time points out of the seven to belong to 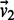, at Day 1 to belong to 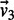 and at Day 91 to belong to 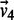. This lead to the identification of eight groups of motifs, i.e. two for each characteristic profile (Supplementary Table S1).

**Figure 4.**
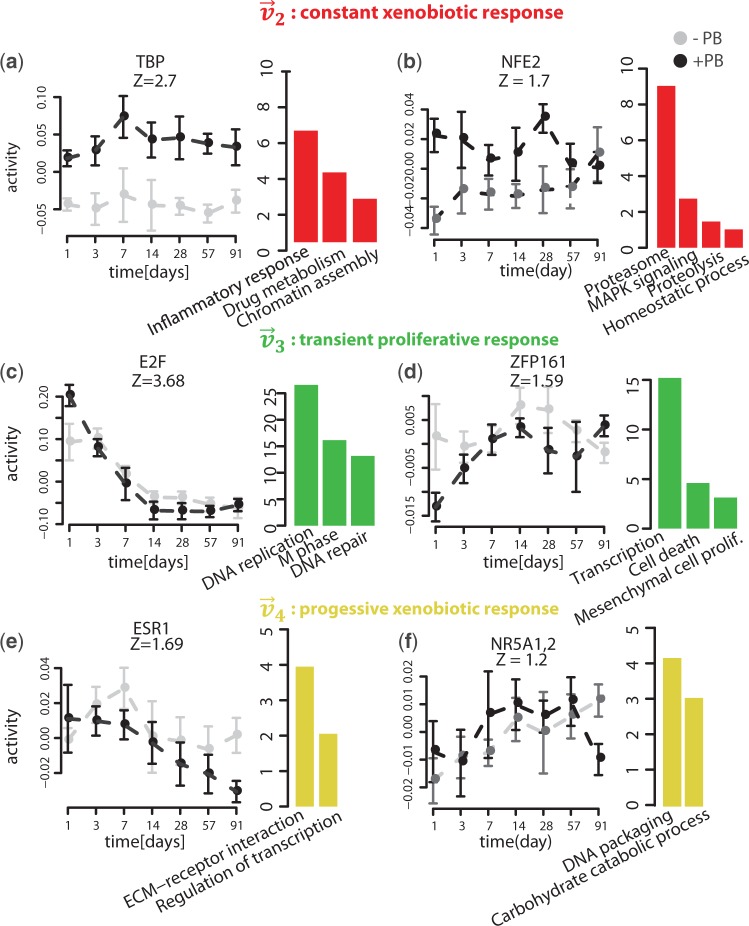
Examples of motif activity profiles that are associated with the constant xenobiotic response (**a,b**), the transient proliferative response (**c,d**) and the progressive xenobiotic response (**e,f**). For each motif, a *z*-value is indicated that quantifies the overall significance of the motif in the early kinetic dataset. In addition, for each motif a selection of biological pathways and functional categories (Gene Ontology or KEGG) that are enriched among target genes is plotted to the right of the activity profile. The size of each bar corresponds to the significance 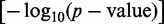 of the enrichment.

To further investigate the biological roles of the motifs associated with the four singular vectors, we performed Gene Ontology and KEGG functional enrichment analysis of the targets of the eight groups of motifs that are either positively or negatively associated with one of the singular vectors (Figure 4 and Supplementary Figure S5). Below is a brief description of most important findings.

##### Constant xenobiotic response

As discussed further below, it is well known that CAR is a crucial regulator involved in the xenobiotic response and thus a prime candidate for a regulator associated with a constant xenobiotic response. Unfortunately, as there is currently no high-quality regulatory motif available for CAR, our TFBS predictions do not include CAR target sites and our analysis is thus unable to infer CAR’s activity *ab initio*. However, our analysis identified several additional regulators that are associated with a sustained xenobiotic response, i.e. a constant difference in activity between the PB-treated and control samples (a full list of associated motifs is presented in Supplementary Table S1).

Among these is TBP, whose targets are significantly upregulated under PB treatment and enriched in oxidation-reduction processes (Figure 4a), and NFE2 whose target genes are involved in homeostatic processes (Figure 4b) and include the proteasome complex (e.g. *Psmc3*, *Ufd1l* and *Ube2v1*) and oxidative stress genes (e.g. *Ggt1*, *Txn1* and *Adh7*). These targets represent key pathways of the liver drug-induced response that have been recently shown to be regulated by NFE2 in hepatocytes (49).

##### Transient proliferative response

It has been observed previously that PB treatment leads to a transient mitogenic response (18,28,20). Our analysis revealed that the process is positively regulated by the E2F family of TFs, whose motif activity is significantly increased at Day 1 upon PB treatment (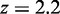). E2F family members are known regulators of cell proliferation and the functions of their predicted targets (Figure 4c) further confirms their specific role in DNA replication, DNA repair and mitosis (50–53). Interestingly, while eight TFs are potentially binding this motif, three of them (E2F1, E2F2 and E2F8) display a positive correlation between their gene expression and the motif activity in the time course (Supplementary Figure S3a and Supplementary Table S6), suggesting that it may be these three TFs that are involved in the PB-mediated transient hyperplastic response.

Our analysis predicted ZFP161 as an additional regulator of the transient hyperplastic response, whose targets are downregulated upon PB treatment. Interestingly, ZFP161’s target genes are enriched in transcriptional repressors (e.g. *Rb1*, *Bcl6*, *Tle2*, *Klf9* and *Foxp1*), many of which are known to repress the cell cycle and cell growth. Moreover, positive regulation of cell proliferation by ZFP161 is further supported by negative regulation of cell death genes (Figure 4d). Together, these results suggest that PB-mediated ZFP161 activation may lead to the downregulation of an important group of transcriptional repressors and concomitant cell cycle activation.

##### Progressive xenobiotic response

Finally, our analysis identified several motifs associated with a divergence between motif activity in the PB-treated and control samples in the last month of the time course. Among the downregulated motifs is ESR1, whose predicted targets regulate extracellular matrix (ECM) genes and may thus regulate tissue remodeling (Figure 4e). NR5A1,2 is an additional regulator whose activity was downregulated after 3 months of PB treatment (Figure 4e). Interestingly, Nr5a2 (known as liver receptor homolog-1 or LRH-1) is an established regulator of cholesterol, bile acid homeostasis, glucose and lipid metabolism (54,55), as confirmed by predicted targets functions in carbohydrate metabolism (Figure 4f).

### Regulators of PB-mediated long-term liver gene expression changes are downstream of CAR signaling

In order to assess the importance of CAR in the livers in physiological conditions, i.e. without PB treatment and to identify to what extent the response to PB treatment is downstream of CAR activation, we made use of gene expression profiles from CAR WT and KO mice (19). We first identified regulators that are downstream of CAR under physiological conditions by comparing motif activities between non-treated CAR KO and WT samples (Figure 2a provides a schematic representation of all motif activity contrasts that we calculated). Only five motifs were significantly downregulated in their activity upon CAR deletion (Supplementary Table S2 provides a full list). To assess the CAR dependence of the regulatory motif changes mediated by PB treatment, we compared regulatory motifs that are perturbed in activity upon PB treatment in WT animals, with motifs that are perturbed upon PB treatment in CAR KO animals. Strikingly, of the 23 motifs dysregulated upon PB treatment in WT mice, none was dysregulated in KO mice, indicating that all regulators of PB-mediated gene expression changes at Day 161 are downstream of CAR signaling (Supplementary Table S2). This result is in line with previous studies where CAR was shown to be critical for both the acute (20) and chronic (19) transcriptional response to PB treatment. These results are further confirmed by an SVD analysis (Supplementary Results and Supplementary Figure S6), which shows that the major source of motif activity changes in these liver samples is the CAR-dependent liver response to PB treatment. In summary, it is highly likely that all motif activity changes observed in the early response time course also depend on CAR activation.

### Identification of specific regulators of promoted tumors involved in early PB-mediated response

Our analysis above has focused on regulators that are perturbed during the first 3 months of PB treatment, whereas it takes several more months for tumors to be detected at the histopathologic level (21). We next investigated which regulators have different activities in the end-stage tumors that are observed after 8 months of treatment, as compared with their surrounding tissue (Figure 2b). We hypothesized that motifs perturbed both in the early response as well in the end-stage tumors may likely be involved in the process of tumor formation. Moreover, we distinguished ‘promoted’ tumors, which are characterized by mutations that cause constitutive activation of β-catenin, from ‘non-promoted’ tumors that are characterized by mutations in Ha-ras activation. Motifs that are perturbed in promoted tumors, but not in Ha-ras tumors, are prime candidates for involvement in the non-genotoxic tumor promotion.

**Figure 5.**
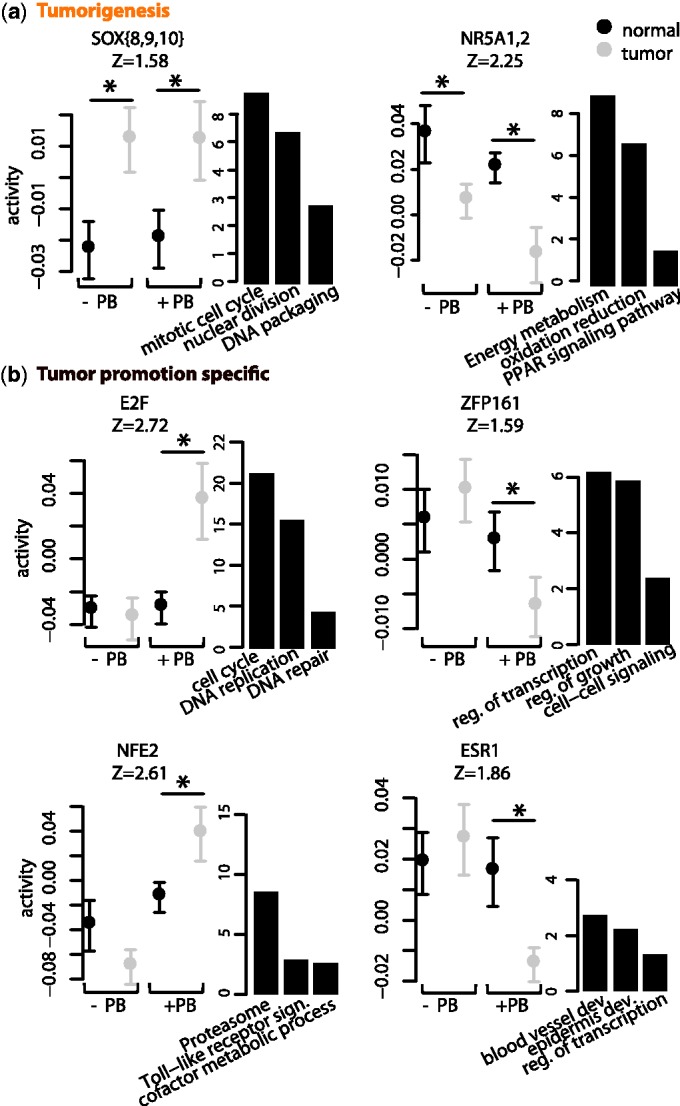
Regulators of liver tumorigenesis and tumor promotion. (**a**) Activities of two regulators that are dysregulated in both promoted and non-promoted tumors. (**b**) Activities of four regulators that are specifically dysregulated in promoted tumors. For each regulator, the activities in the tumor and surrounding normal tissue are indicated by black and turquoise points, respectively. A *z*-value quantifying the overall significance of the motif in tumor dataset is indicated below each motif’s name. A selection of biological pathways and functional categories (Gene Ontology or KEGG) enriched among target genes of these motifs are shown on the right of each activity profile. The height of each bar corresponds to the significance [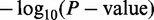] of the enrichment. Differences in activity between the tumor and surrounding tissues that are significant are indicated by an asterisk (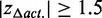).

We find eight motifs that are perturbed in both promoted and non-promoted tumors (Supplementary Table S3). Half of these were also associated with one of the singular vectors of the early PB treatment time course. In particular, the motif NR5A1,2 was associated with singular vector 4, showing a downregulation in the PB-treated animals in the third month of the time course, is also downregulated in the end-stage tumors. Predicted targets for NR5A1,2 are involved in several known metabolic functions of the liver [oxido-reduction processes, peroxisome proliferator-activated receptor (PPAR) signaling, and energy metabolism] (55), consistent with target functions at the early time points, indicating that NR5A1,2 downregulation is associated with hepatocyte loss of function (Figure 5a). Furthermore, our analysis identifies SOX{8,9,10} as a regulator of cell proliferation in both promoted and non-promoted tumors (Figure 5a). As these motifs are perturbed in both promoted and non-promoted tumors, they likely regulate genes involved in general liver tumor biology and are presumably not relevant for the specific process of non-genotoxic tumor promotion.

Seven motifs were dysregulated in promoted tumors only (Supplementary Table S4). Strikingly, all but one of these motifs were associated with one of the singular vectors of the early PB treatment time course. In particular, the E2F motif, which we found to be a positive regulator of both the postnatal liver growth and the transient PB-mediated mitogenic response, is here observed to be upregulated only in promoted tumors (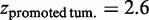), showing no significant perturbation in the non-promoted tumors (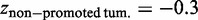). Importantly, E2F1, E2F2 and E2F8, previously identified as strong candidate regulators of early PB-mediated transient hyperplastic response, display similar positive correlation between their gene expression and the motif activity in the tumor (Supplementary Figure S3a and Supplementary Table S5). Notably, the cellular functions regulated by SOX{8,9,10} and E2F at tumor stage (Figure 5a and b) suggests that these motifs have distinct regulatory effects on cell proliferation; while SOX{8,9,10} regulates mitosis, E2F targets specifically regulate DNA replication (Supplementary Figure S2).

The ZFP161 motif, which we found to negatively regulate transcriptional repressors of the cell cycle in the early stages of PB treatment, also displays significant decrease in activity in promoted tumors (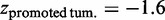), but not in non-promoted tumors (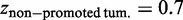) (Figure 5b). Interestingly, these results suggest that similar regulatory mechanisms, involving E2F and ZPF161, are responsible for the proliferation that occurs transiently immediately upon PB treatment as well as the proliferation in promoted tumors. Moreover, this upregulation of proliferation, which might involve the release of specific cell-cycle check-points, is clearly distinct from the regulatory mechanism responsible for upregulation of proliferation in the non-promoted tumors.

Another motif specifically upregulated in promoted tumors is NFE2 (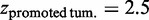 versus 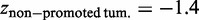). Furthermore targets of NFE2 that already showed upregulation at the early stage, such as several members of the protease family and oxidative stress response, e.g. *Aox1*, *Acox2*, *Srxn1* and *Mocos*, show continued activation in the promoted tumors (Figure 5b).

Finally, our analysis revealed a significant decrease in activity of the motif bound by ESR1 in promoted tumors only (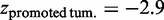 versus 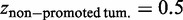). Moreover, our analysis shows ESR1 regulation of genes involved in anatomical structure morphogenesis/tissue remodeling (genes, e.g. coding for collagen and fibronectin) that are progressively downregulated upon PB treatment and remain repressed at the tumor stage (Figure 5b). We also performed an SVD analysis of the activity matrix of this dataset (Supplementary Results and Supplementary Figure S7). The analysis identified the most significant singular component with regulators of promoted tumors that largely overlap those identified by differential motif activity analysis. The second singular component identified a number of regulators of liver tumorigenesis. Interestingly, these motifs were not identified by differential motif activity analysis, suggesting that SVD analysis can identify a significant effect of a set of motifs even when the differential activity of each motif is not significant by itself.

### Early regulators of liver tumor promotion downstream of β-catenin signaling

It has been established that liver tumor promotion by PB requires functional β-catenin (56) and promoted tumors are characterized by mutations that cause constitutive activation of β-catenin. However, it remains unclear how PB promotes the outgrowth of pre-existing β-catenin activated cells. The ability for β-catenin to physically interact with various co-factors and nuclear receptors (57,58) suggests that the predicted regulators of PB-mediated liver tumor promotion may interact with the β-catenin pathway.

We thus investigated which regulators are downstream of β-catenin under physiological conditions by comparing motif activities in non-treated WT and β-catenin KO cells (Figure 2c). This analysis showed massive changes in regulatory activities upon KO of β-catenin, with as many as 33 motifs significantly perturbed in their activity (Supplementary Table S5). Note that this analysis successfully retrieved known co-factors of β-catenin such as the Tcf7-Lef1 motif, whose activity decreases strongly upon β-catenin KO (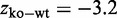). Furthermore, two of the previously identified regulators of liver tumor promotion, i.e. E2F (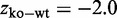) and NFE2 (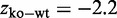), were negatively modulated upon β-catenin KO, whereas ESR1 (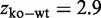) was positively modulated. These findings support the hypothesis of a positive interaction between E2F/NFE2 and the β-catenin signaling pathway. The strong positive correlation between ESR1 gene expression and motif activity in both this study and the tumor study (Supplementary Figure S3 and Supplementary Table S6) supports a negative interaction between the β-catenin signaling pathway and ESR1 in liver, potentially through direct repression of target gene by β-catenin.

## DISCUSSION

Here we describe a novel bioinformatics approach for the automated identification of independent transcription regulatory programs within a complex *in vivo* tissue environment. Using well-characterized mouse mechanistic models for non-genotoxic hepatocarcinogeneis, we were able to successfully infer the contributions of key regulators of phenobarbital-mediated xenobiotic responses, tumor promotion and end-stage tumors as well as assess their dependence on the CAR and β-catenin signaling pathways.

Motif activity response analysis, which models observed gene expression patterns in terms of computationally predicted TF-binding sites, has been specifically designed to identify the key regulators responsible for the observed gene expression dynamics. One of its strengths is that MARA does not rely directly on the mRNA expression of the TFs, but instead infers the activities of regulators from the expression of their predicted target genes. Consequently, MARA can easily identify changes in motif activities that are due to post-translational modifications, changes in cellular localization or interactions with co-factors. This is specifically relevant for our model system in which PB indirectly triggers changes in gene expression via EGFR signaling-mediated post-translational modification and nuclear translocation of the TF CAR (15,16).

A major challenge in the analysis of the complicated *in vivo* systems such as the one we study here, is that the observed genome-wide expression changes result from multiple biological pathways dynamically changing in parallel. Consequently, even when MARA allows us to infer the regulatory activities of key TFs across the samples, it may be challenging to identify the independent biological processes that these regulators contribute to and how each regulator is contributing to each process. To address this, we here developed a new analysis approach based on SVD that decomposes the entire matrix of motif activities across all samples and identifies the major mutually independent activity profiles.

**Figure 6.**
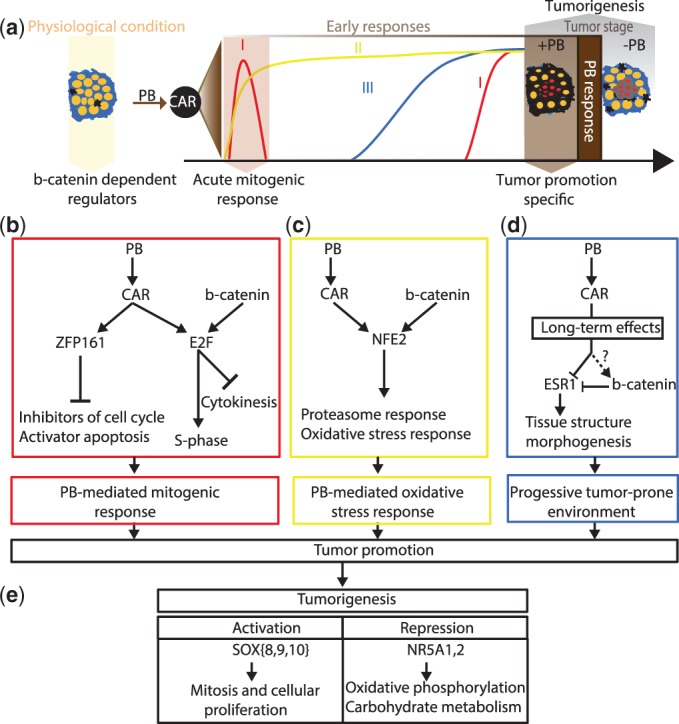
Schematical representation of PB-mediated tumor promotion, as it emerges from our study. (**a**) Illustration of the PB-mediated tumor promotion process and the aspects elucidated by the four experimental studies that we analyze. KO of β-catenin identifies regulators downstream of β-catenin in physiological conditions (yellow arrow). This study and previous analyses suggest that all regulatory effects of PB treatment are downstream of CAR activation (brown arrow and black circle). This study’s motif activity and SVD analysis of the early kinetic time course identified three key biological processes induced by PB treatment: a transient mitogenic response, which is also associated with a late resurgence of proliferation (I, red), a sustained xenobiotic response (II, yellow) and a late response which is likely involved in establishing a tumor-prone environment (III, blue). Comparison of promoted and non-promoted tumors identifies motifs dysregulated in all tumors and in promoted tumors only (gray arrows). (**b–d**) Summary of the key regulators of liver tumor promotion organized according to biological processes (colored boxes matching the colors of processes I, II and III in panel a) with arrows indicating regulatory interactions between regulators and on selected target genes. (b) E2F and ZFP161 regulate PB-mediated hepatocyte proliferation at the early and promoted tumor stage. E2F is downstream of β-catenin signaling and likely induces both DNA replication, via upregulation of *E2f1,2* and aborted cytokinesis via upregulation of *E2f8* and *c-myc*. ZFP161 is likely involved in the G0–G1 transition via transcriptional repression of transcriptional repressors of cell growth and cell cycle. (c) NFE2, downstream of β-catenin as well is involved in the sustained xenobiotic response, upregulating proteasome activity and the oxidative stress response. (d) PB-mediated suppression of ESR1 activity underlies development of a tumor-prone environment, most likely through repression of tissue morphogenesis. β-Catenin signaling represses ESR1. (**e**) Key regulators involved in tumorigenesis, i.e. dysregulated in both promoted and non-promoted tumors. Increased SOX{8,9,10} activity likely regulates hepatocyte mitosis and proliferation via upregulation of cyclins. Decrease in NR5A1,2 activity is detected after 3 months of PB treatment and maintained in tumor samples and therefore a good early indicator of hepatocyte loss-of-function associated with tumorigenesis.

Our results show that this approach successfully identifies the major biological pathways underlying the response to PB treatment and it furthermore allows us to identify how the key regulators are contributing to each of these pathways. We identified the roles of E2F and ZFP161 in the regulation of cell proliferation in both the early transient mitogenic response and specifically in promoted tumors. We identified ESR1 as a key regulator of establishing a tumor-prone environment and we identified NFE2 as a key regulator of the sustained xenobiotic response. Figure 6 schematically summarizes these key findings, showing both the overall picture that emerges of the biological processes involved in PB-mediated tumor promotion (Figure 6a) as well as the key regulators that we identified and their role in the various processes (Figure 6b).

In the next sections we discuss these key findings, put them into context of relevant available literature and put forward concrete hypotheses for the biological mechanisms involved in these regulatory processes. Finally, where possible, we also discuss pieces of supporting evidence for the hypotheses we put forward.

### E2F as a positive regulator of the PB-mediated proliferative response at both the early and tumor stages

An important aspect of PB-mediated tumor promotion is the ability of PB to induce a transient mitogenic response and to cause liver neoplasia upon chronic administration. However, the exact mechanisms responsible for the exit from the quiescent state and the re-entry into the cell cycle remain largely unknown [see (59) for a review]. Our analysis revealed that the regulatory motif bound by the E2F family of TFs is one of the key factors positively contributing to the early proliferative response upon PB treatment. In addition, E2F is upregulated in promoted tumors, but not in non-promoted tumors. Importantly, the absence of E2F motif modulation in non-promoted tumors argues against the hypothesis that the motif is simply reflecting increased proliferative activity. Furthermore, the fact that KO of β-catenin in physiological conditions leads to downregulation of E2F activity implies that β-catenin positively regulates E2F activity (either directly or indirectly) and suggests that PB-mediated activation of β-catenin may contribute to the upregulation of E2F activity at the tumor stage.

The plausibility of a role for E2F TFs in PB-mediated tumor promotion is supported by numerous studies reporting a central role of distinct E2F family members in hepatocellular carcinoma (60,61). More specifically, PB-mediated modulation of E2F gene regulation in freshly isolated hepatocytes has been previously suggested (62). Here we show a highly specific upregulation of E2F activity in promoted tumors and a potential role in tumor promotion through β-catenin-mediated activation.

The E2F family contains eight different TFs that can bind to the E2F motif and the MARA analysis does not directly predict which of these eight TFs is mainly responsible for the activity of the E2F regulatory motif in this system. However, measurements of motif activity correlation with mRNA expression of the TFs (Supplementary Figure S4 and Supplementary Table S6) shows that the expression of E2F1, E2F2 and E2F8 exhibit the most significant correlation with E2F motif activity in the time course and tumor studies. This makes these TFs the most likely candidates for driving the E2F motif activity, but it should be noted that motif activity changes do not necessarily require changes in mRNA levels of the binding TFs, i.e. the activity change may be due to post-translational modifications, nuclear localization, etc. E2F7 and E2F8 have been recently shown to play a key role in positively regulating hepatocyte polyploidy (63,64). Interestingly, Myc has been shown to be an additional positive regulator of polyploidy in hepatocytes (65,66). Furthermore, both *E2f8* and *c-myc* are significantly upregulated in promoted tumors only and both are predicted targets of E2F. Given that ligands of nuclear receptors such as PB and TCPOBOP have been shown to cause liver polyploidization (59,67,68), we propose that both E2F1 and E2F8 are responsible for the E2F activity modulation at the tumor stage and that they regulate distinct cell cycle checkpoints, in particular, regulation of entry in S-phase for E2F1 and inhibition of cytokinesis for E2F8 together with Myc (Figure 6b).

### ZFP161 as transcriptional repressor involved in the PB-mediated proliferative response at both the early and tumor stages

Our analysis revealed a decrease in activity of the motif bound by ZFP161 (also known as ZF5), i.e. an overall downregulation of its predicted targets upon PB treatment contributing to the early transient proliferative response. In addition, ZFP161 targets are downregulated in promoted tumors, but not in non-promoted tumors. Affymetrix gene expression analysis shows that while ZFP161 is not transcriptionally regulated by PB and its mRNA expression is not correlated with motif activity (Supplementary Figure S3), it is clearly expressed in the liver (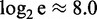).

Although ZFP161 has been shown to be preferentially active in differentiated tissues with little mitotic activity (69), where it was shown to act as a transcriptional repressor of *c-myc* (70,71), we here show an increase in ZFP161 transcriptional repression of target genes enriched in transcriptional repressors (i.e. *Mxi1* and *Klf10*), several of these being negative regulators of cell cycle and cell growth. Therefore, we hypothesize that ZFP161 participates in the PB-mediated regulation of quiescent hepatocyte G0–G1 transition at both the early and tumor stages, by repressing negative regulators of cell cycle and positive regulators of apoptosis (Figure 6b).

### The progressive PB-mediated downregulation of ESR1 contributes to establishing a tumor-prone environment

PB-mediated tumorigenesis involves dynamic changes in tissue composition, and the adaptive response of the liver to chronic stress eventually leads to the establishment of a tumor-prone environment. The identification of key factors that contribute to this process could provide valuable insight into the development of PB-mediated tumorigenesis. Our analysis identified ESR1 as a factor progressively downregulated upon chronic PB exposure, starting in the third month of PB treatment. In addition, ESR1 activity is downregulated in promoted tumors, but not in non-promoted tumors. These two observations make ESR1 a strong candidate regulator for the process of establishing a tumor-prone environment. Furthermore, β-catenin KO in physiological conditions leads to upregulation of ESR1 activity, implying that β-catenin represses (directly or indirectly) ESR1. Further supporting this direct link between β-catenin and ESR1 repression is the fact that the highest correlations between ESR1 activity and mRNA expression levels are observed in the β-catenin KO and tumor studies, i.e. precisely those experiments where β-catenin activity is predicted to change (Supplementary Figure S3). Importantly, a physical interaction between β-catenin/TCF-4 and ESR1 has already been reported in other physiological contexts (72,73). That ESR1 can have tumor suppressor activity is supported by various studies (4,74–77). However, here we propose more specifically that the progressive suppression of ESR1 activity from early hyperplastic tissue to cancer (78) is mediated by PB chronic exposure and is one of the mechanisms underlying PB-mediated liver tumor promotion due to negative regulation of tissue morphogenesis (Figure 6d).

### NFE2 as a regulator of exacerbated xenobiotic response associated with promoted tumors

Our analysis revealed that PB treatment causes a constant upregulation of homeostatic processes via NFE2 activation of proteasome and oxidative stress biological processes during the early phases of treatment and that this upregulation persists into promoted tumors (Figure 6c). Of note, NFE2 regulatory activity in homeostatic processes has been shown in a recent study (49). This upregulation of NFE2 in tumors compared to the surrounding tissue is specific to promoted tumors. Furthermore, the fact that NFE2 activity is downregulated upon β-catenin KO in physiological conditions strongly suggests that β-catenin signaling is positively regulating NFE2 activity. As β-catenin is also involved in the regulation of drug metabolizing enzymes in the liver (79–82), we hypothesize that NFE2 and β-catenin cooperate in regulating genes involved in drug metabolism and that the xenobiotic response is partly exacerbated in promoted samples upon constitutive activation of β-catenin, resulting in further upregulation of NFE2.

### Regulators of liver tumorigenesis

Our analysis identified several regulators of liver tumorigenesis (Supplementary Table S3). Interestingly, NR5A1,2 downregulation is observed early in the process of tumor promotion (after 3 months of PB treatment). Given its apparent role in hepatocyte liver function regulation (Supplementary Figure S1) we hypothesize that NR5A1,2 is associated with hepatocyte loss of function (Figure 6e). SOX{8,9,10} is an additional regulator of liver tumorigenesis and our analysis indicates a role in hepatocyte proliferation. Finally, comparing functional enrichment between the target genes of SOX{8,9,10} and E2F at tumor stage revealed that while E2F specifically regulates DNA replication (Supplementary Figure S2), SOX{8,9,10} preferentially targets mitotic genes (Supplementary Figure S1). These results support our hypothesis that E2F targets cell cycle check points that are distinct from those shared with other tumors.

### Future extensions of the modeling approach

In future work we will aim to address several limitations of the current modeling approach. First and foremost, the method is currently limited to inferring the activities of only those TFs for which sequence specificities are known, i.e. roughly 350 of the approximately 1500 mouse TFs. For example, we were not able to predict CAR motif activity as there is, to our knowledge, no high quality sequence motif available for CAR. This is not an intrinsic limitation of the method and as regulatory motifs for an increasing number of TFs becomes available, they can easily be incorporated into the method.

Another major limitation of MARA is that it currently focuses solely on predicted TFBSs in proximal promoters, ignoring the effects of distal enhancers. Although a number of combined experimental and computational methods have been put forward recently that allow genome-wide mapping of active enhancers [e.g. (83)], these methods require considerable investment and enhancer maps are only available for a small set of selected model systems. As the locations of relevant enhancers vary highly across tissues and model systems, successful incorporation of enhancers into MARA requires the availability of enhancer maps for the specific system under study.

Most importantly, all the hypotheses discussed in this work are based on analysis of high-througput data and future experimental studies will be required to characterize our inferred TF activities in more detail at the biochemical level. Such studies may include chromatin immunoprecipitation (ChIP) assays on liver tissue from control and phenobarbital-treated mice.

## CONCLUSION

We have demonstrated that by combining motif activity response analysis with SVD, we are able to automatically untangle the regulatory activities underlying the perturbation of multiple biological pathways in complex *in vivo* systems and derive novel hypotheses regarding the key regulators and their role in the process. Our analyses provide novel mechanistic insight for PB-mediated tumor promotion in the mouse liver, including the identification of E2F and ZFP161 as regulators of PB-mediated hepatocyte proliferation at both early and tumor stages and progressive PB-mediated suppression of ESR1 activity that may contribute to the development of a tumor-prone environment. These findings may also help identify novel biomarkers for assessing the carcinogenic potential of xenobiotics.

## SUPPLEMENTARY DATA

Supplementary Data are available at NAR Online, Supplementary Material and Methods, Supplementary Results, Supplementary Figures S1–S7, Supplementary Tables S1–S7 and Supplementary References 1–20.

## FUNDING

Innovative Medicine Initiative Joint Undertaking (IMI JU) (115001) (MARCAR project; http://www.imi-marcar.eu/); Novartis. EvN was supported by the Swiss Systems Biology Initiative SystemsX.ch within the network “Cellplasticity”. Funding for open access charge: University of Basel.

*Conflict of interest statement*. None declared.

## Supplementary Material

Supplementary Data
