# Supplementary material for: Computational modeling identifies key gene regulatory interactions underlying phenobarbital-mediated tumor promotion
Source: Nucleic Acids Res. 2014 Jan 23;42(7):4180–95. doi: 10.1093/nar/gkt1415 (PMC3985636; doi:10.1093/nar/gkt1415)
Supplement: Supplementary Data [file supp_42_7_4180__index.html]

Computational modeling identifies key gene regulatory interactions underlying phenobarbital-mediated tumor promotion — Computational modeling identifies key gene regulatory interactions underlying phenobarbital-mediated tumor promotion — Supplementary Data 

# Computational modeling identifies key gene regulatory interactions underlying phenobarbital-mediated tumor promotion

## Supplementary Data

files

**Files in this Data Supplement:**

- Supplementary Data - pdf file
