# Supplementary material for: Computational modeling identifies key gene regulatory interactions underlying phenobarbital-mediated tumor promotion
Source: Nucleic Acids Res. 2014 Jan 23;42(7):4180–95. doi: 10.1093/nar/gkt1415 (PMC3985636; doi:10.1093/nar/gkt1415)
Supplement: Supplementary Data [file supp_gkt1415_nar-02881-n-2013-File012.pdf]

Supplementary Data

Raphaëlle Luisier [1], Elif B. Unterberger [2], Jay I. Goodman [3], Michael Schwarz [2], Jonathan Moggs [1], Rémi Terranova [1,*] and Erik Van Nimwegen [4,*]

[1]Discovery and Investigative Safety, Novartis Institutes for Biomedical Research, 4057 Basel, Switzerland

[2]Department of Toxicology, Institute of Experimental and Clinical Pharmacology and Toxicology, University of Tübingen, 72074 Tübingen, Germany

[3]Department of Pharmacology and Toxicology, Michigan State University, 48824 Michigan, U.S.A.,

[4]Biozentrum, University of Basel, and Swiss Institute of Bioinformatics, 4056 Basel, Switzerland

[*]To whom correspondence should be addressed: remi.terranova@novartis.com, erik.vannimwegen@unibas.ch

December 19, 2013

1 Supplementary Material and Methods

1.1 Gene expression datasets

A library of 109 genomewide mRNA expression patterns was compiled from four different studies (**Figure 1a**): 70 samples from a time series of expression data from liver samples of B6C3F1 vehicle- (i.e. control) or PB-treated mice at +1, +3, +7, +14, +28, +57 and +91 days of dosing (5 replicates) [1]; 8 mRNA expression patterns in livers of wild-type and hepatocyte-specific β-catenin knockout C3H/N [2] animals; 13 mRNA expression patterns in livers of wild-type and CAR knock-out C3H/N animals DEN-initiated at 5 weeks of age prior to 23 weeks of PB -or vehicle-treatment [3]. Datasets on global mRNA expression patterns (18 samples) from liver tumors and corresponding surrounding normal tissue of C3H/N animals DEN-initiated at 4 weeks of age prior to 35 weeks of PB- or vehicle-treatment were available to us from IMI-MARCAR partners (Unterberger *et al,(2013)*, manuscript submitted). Screening the tumors for mutations in Ha-ras, B-raf and Ctnnb1 (i.e. the β-catenin coding gene) confirmed that promoted tumors (from animals exposed to PB) were mutated in Ctnnb1 while non-promoted tumors were mutated in Ha-ras (data not shown, Unterberger *et al*, 2013). In all four studies gene expression was profiled using Affymetrix GeneChip MOE-4302 (Affymetrix, Santa Clara, CA) containing approximately 43,000 probe sets.

1.2 Affymetrix GeneChip processing

The analysis of the micro-array data was done with the R statistical package version 2.13 (2005) and Bioconductor libraries version 1.4.7 [4]. The four original data-sets containing Affymetrix CEL files were normalized independently using the Robust Multichip Average (RMA) implementation of the algorithm available in R/Bioconductor [4], producing four expression matrices, and the quality of the experiments was assessed using diverse statistics implemented in the package arrayQualityMetrics for R/Bioconductor [5].

2 Supplementary Results

2.1 Regulators associated with termination of developmental liver growth ($\vec{v}_1$)

To determine motifs underlying the four characteristic modes identified in this study, we selected motifs which contributed and correlated the most with each of the four singular vectors (**Figure 3c,d,e,f**). In this way we obtained, for each of the 4 singular vectors, two clusters of motifs with similar activity profiles, i.e. one correlating negatively with the singular vector, and one correlating positively (**Figures 3d,f**). We further refined the selection of the motifs associated with first singular vector as follows: 1) removing motifs for which the overall significance was lower than $z < 1.5$ and 2) removing motifs whose cognate TFs were not expressed in the liver (log-expression less than 6.0) $\log_2 e \leq 6.0$). This lead to the identification of 6 motifs motifs (**Supplementary Table S1**).

As originally observed in [1], completion of the post-natal liver development process occurs during the early PB-treatment time course, consisting in both hepatocyte proliferation at early stage, and progressive induction of liver-specific genes [6, 7]. We here identify key regulators of these two processes: 1. we show that post-natal liver growth (that decreases over time) is regulated by known regulators of cell proliferation such as the E2F family of TFs [8, 9, 10], SRF [11] and Myc [12, 13]; predicted target genes of these motifs have functions related to cell cycle and DNA replication (**Supplementary Figure S6i**), confirming the role of these regulators in cell proliferation. 2. We show that post-natal liver differentiation (which increases over time) is partly regulated by AHR, a known regulator of drug-metabolizing genes and transporters [14, 15, 16, 17] that has been shown to play key role in liver development [18]. Thus, the main biological process associated positively with the first singular vector is cellular proliferation associated with post-natal liver growth for the first two weeks of the time course. Conversely, the targets of the motifs that are negatively associated with the first singular vector, i.e. corresponding to genes that increase their expression after the first two weeks, are enriched for functions associated with hepatocyte terminal differentiation, such as 'liver development', 'drug metabolism' and 'transcriptional regulation'.

2.2 Singular value decomposition analysis of the activity matrix of the CAR KO data-set

In order to identify and quantify the sources of motif activity changes in the CAR KO data-set, we performed Singular Value Decomposition (SVD) of the activities of the 189 motifs across the four conditions (PB- and vehicle treated livers from wild-type and CAR KO mice). Over 50% of the variance in the activity matrix was explained by the first two components of the SVD as evidenced by the spectrum of singular values (**Figure S6a**).

In order to facilitate the biological interpretation of the singular vectors, we plotted the averaged activities of the right singular vectors v_{ks} over each of the four sample groups and further identified regulatory motifs whose activity profiles correlate most strongly (either positively or negatively) with the activity profile of the singular vector. Visualization of the averaged activity of the first two singular vectors $\vec{v}_1$ and $\vec{v}_2$ in each of the four sample groups is shown in **Figure S6b** and scatter plots of the correlations ρ_i and projections p_i of all motifs i with the first and second right singular vectors are shown in **Figure S6c**.

The first right singular vector accounts for 33% of the variance and is characterized by a positive activity upon PB treatment in wild-type animals only. Given the absence of positive activity in CAR KO treated animals, we propose that this component represents the liver response to PB that is CAR-dependent. Moreover motifs which contribute and correlate most strongly with the first singular vector (TBP, NFE2, REST, GLI1,2,3, FOSL2, ELK1,2, and ZNF143) are all down-stream of CAR signaling under PB treatment (**Table S2**) except CTCF, RXRG-dimer and STAT5{A,B}, further supporting the association of this component with the CAR-dependent liver response to PB treatment.

The second right singular vector accounts for 18% of the variance and is characterized by 1) a lower activity in wild-type liver samples compared to CAR KO samples, and 2) by an activity further lowered upon PB treatment in both wild-type and CAR KO samples (**Figure S6b**). We propose that this component represents the basal liver activity down-stream of CAR that is further exacerbated upon PB treatment. However the motifs that contribute and correlate most strongly with the second singular vector do not coincide with any of the 5 motifs identified by differential motif activity analysis as down-stream of CAR signaling under physiological condition (**Table S2**). Furthermore the average activities have large associated error-bars for each sample group, indicating that the interpretation of this component must be considered with caution.

In conclusion, the SVD-based analysis of the activity matrix of the CAR KO data-set indicates that the major

source of motif activity changes in these liver samples is the CAR-dependent liver response to PB treatment. This result is in line with the analysis based on differential motif activity. Importantly, prior biological knowledge indicates that at least two biological processes are occurring in this system, i.e the CAR KO effect and the xenobiotic response to PB treatment. Differential motif activity previously showed only a very minor CAR KO effect (only 5 motifs identified as down-stream of CAR signaling under physiological condition, see **Table S2**) that may explain the absence of strong association of any component with this biological process.

2.3 Singular value decomposition analysis of the activity matrix of the tumor study data-set

In order to identify and quantify the sources of motif activity changes in the tumor data-set, we performed Singular Value Decomposition (SVD) of the activities of the 189 motifs across the four conditions (PB- and vehicle treated normal and tumorigenic liver samples). Over 57% of the variance in the activity matrix was explained by the first two components of the SVD that are the two significant components of the matrix, as evidenced by the spectrum of singular values (**Figure S7a**).

In order to facilitate the biological interpretation of the singular vectors, we plotted the averaged activities of the right singular vectors v_{ks} over each of the four sample groups and further identified regulatory motifs whose activity profiles correlate most strongly (either positively or negatively) with the activity profile of the singular vector. Visualization of the averaged activity of the first two singular vectors $\vec{v}_1$ and $\vec{v}_2$ in each of the four sample groups is shown in **Figure S7b** and scatter plots of the correlations ρ_i and projections p_i of all motifs i with the first and second right singular vectors are shown in **Figure S7c**.

The first right singular vector accounts for 32% of the variance (**Figure S7a**) and is characterized by 1) a higher activity in PB-treated samples relative to non-treated samples, 2) an increased positive activity in promoted tumor samples relative to all other sample groups (normal treated and non-treated samples, and non-treated tumor samples) and 3) a slight decreased activity in non-promoted tumor samples relative to surrounding normal tissue (**Figure S7b**). Moreover several motifs which contribute and correlate most strongly with the first singular vector (NFE2, E2F1-5, PBX1, and ESR1) as depicted in **Figure S7c**, have been identified as specific regulators of promoted tumors by differential motif activity analysis (see **Table S4**). These results indicate that motifs associated with this component are generally associated with a response to PB treatment which is further 1) exacerbated in promoted tumor samples and 2) inhibited in non-treated tumor samples, suggesting that the first component captures motifs associated with biological pathways underlying promoted tumors that are already up-regulated upon PB treatment and down-regulated in non-promoted tumors.

The second right singular vector accounts for 25% of the variance (**Figure S7a**) and is characterized by an overall decreased activity in tumor samples relative to normal samples, irrespective of the PB treatment (**Figure S7b**); this suggests that the second component captures motifs associated with biological pathways underlying tumorigenesis. It is however noteworthy that none of the motifs which contribute and correlate most strongly with the second singular vector (**Figure S7c**) were identified as regulators of tumorigenesis by differential motif activity analysis (**Table S3**). One explanation for this could be a strong variability in activity profiles leading to low Z-value of differential activity.

In conclusion the SVD-based analysis of the activity matrix allows for the identification of 1) regulators of promoted tumors (first component) which are consistent with those identified by differential motif activity analysis, and 2) regulators of liver tumorigenesis, which were not identified by differential motif activity analysis, potentially due to high noise to signal ratio.

3 Supplementary figures

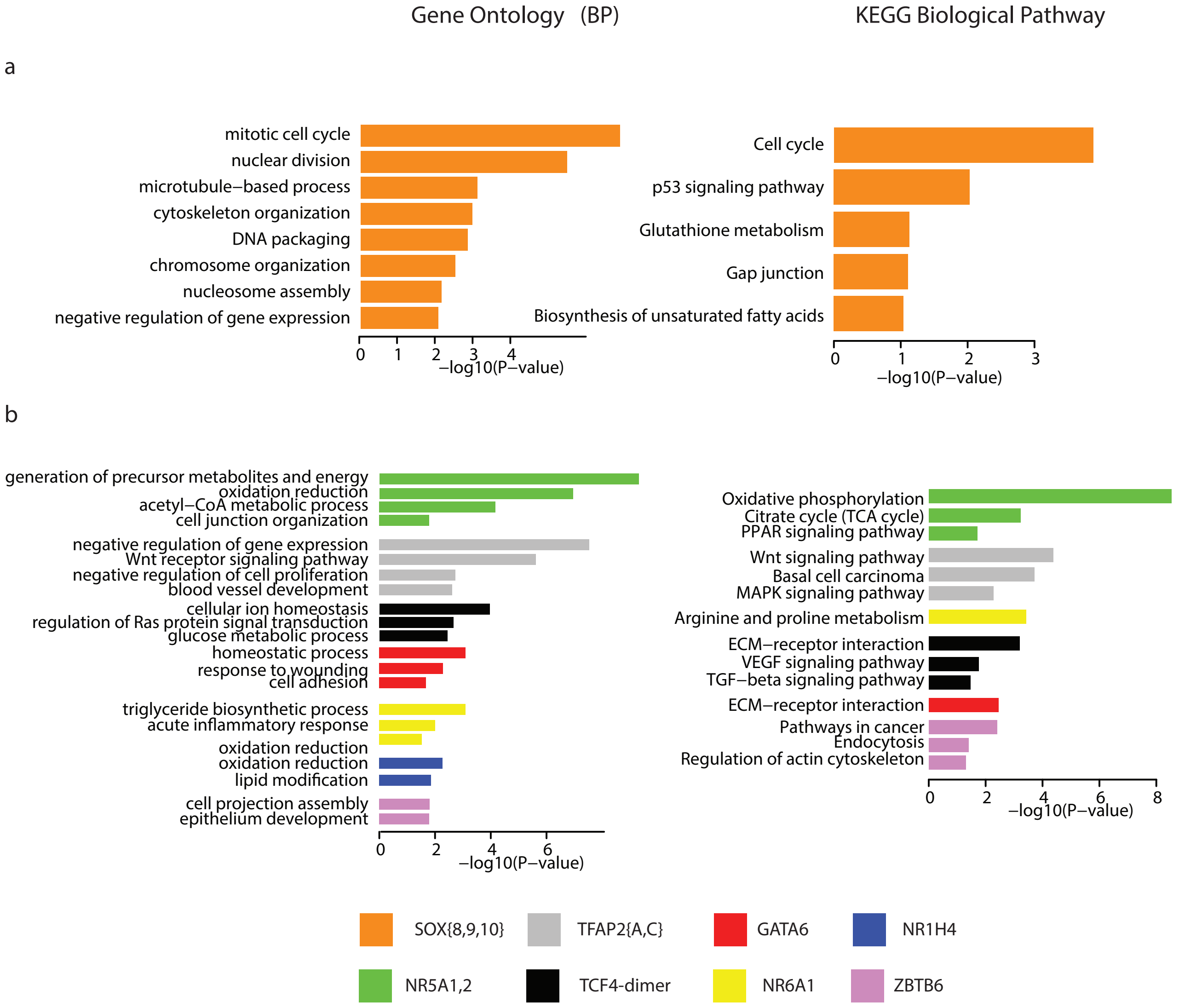

Figure S1: Selection of representative biological terms and processes associated with the predicted target genes of motifs which activities were significantly **(a)** higher or **(b)** lower in promoted tumors relative to surrounding treated normal tissue, and in non-promoted tumors relative to surrounding non-treated normal tissue (**Supplementary Table S3**). Bars are colored according to motif to which the target genes are associated with. Bar height indicates significance of functional enrichment as it represents the $-\log10(P\mathrm{-Value})$ of functional enrichment in the given biological term or process as obtained from the DAVID Bioinformatic Resource (Database for Annotation, Visualization and Integrated Discovery) [19, 20] version 6.7, sponsored by the National Institute of Allergy and Infectious Disease (NIAID), NIH.

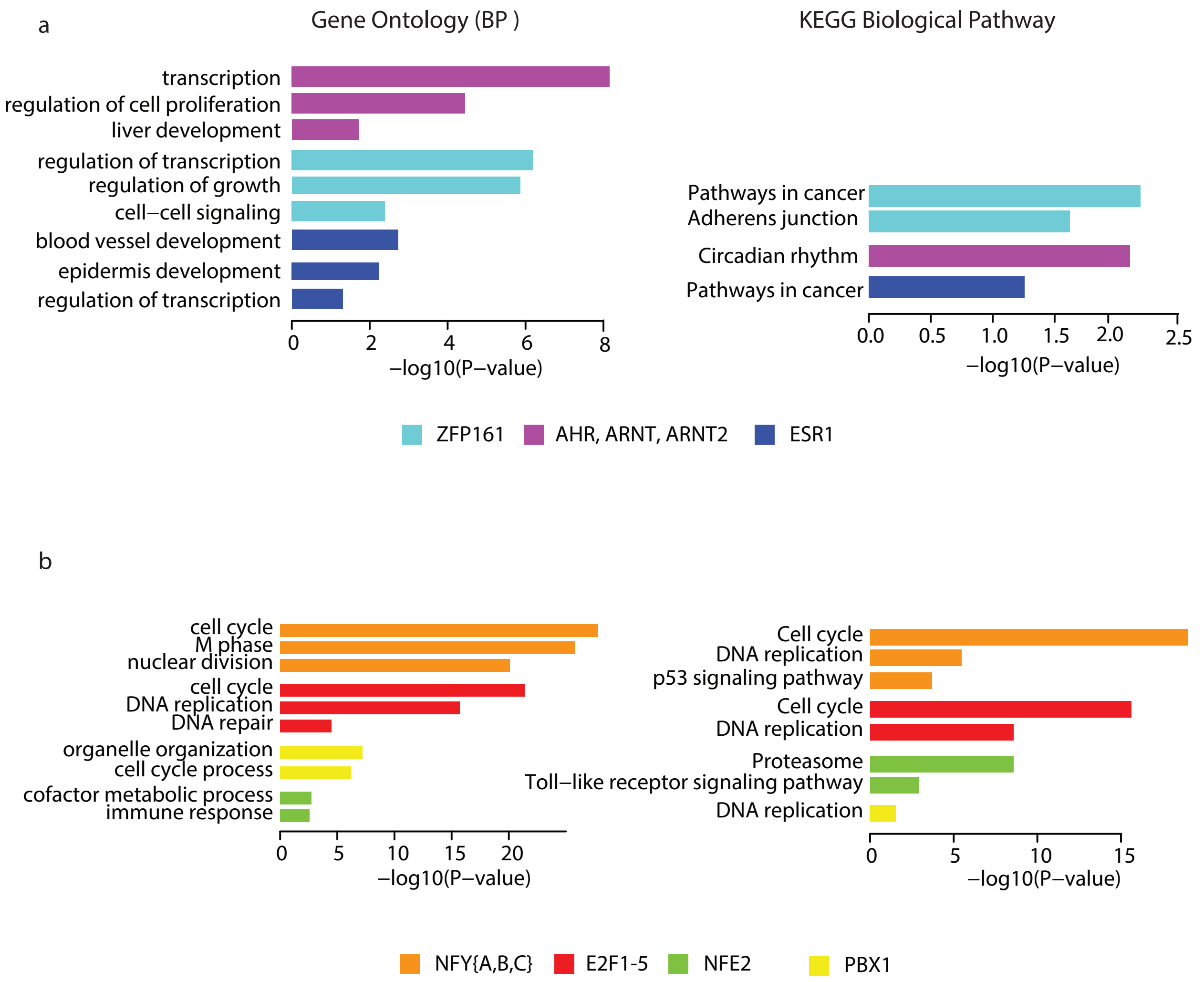

Figure S2: Selection of representative biological terms and processes associated with the predicted target genes of motifs which activity was significantly **(a)** lower or **(b)** higher in promoted tumors relative to surrounding treated normal tissue, but that did not change in non-promoted tumors relative to surrounding non-treated normal tissue (**Supplementary Table S4**). Bars are colored according to motif to which the target genes are associated with. Bar height indicates significance of functional enrichment as it represents the $-\log10(P-\text{Value})$ of functional enrichment in the given biological term or process as obtained from the DAVID Bioinformatic Resource (Database for Annotation, Visualization and Integrated Discovery) [19, 20] version 6.7, sponsored by the National Institute of Allergy and Infectious Disease (NIAID), NIH.

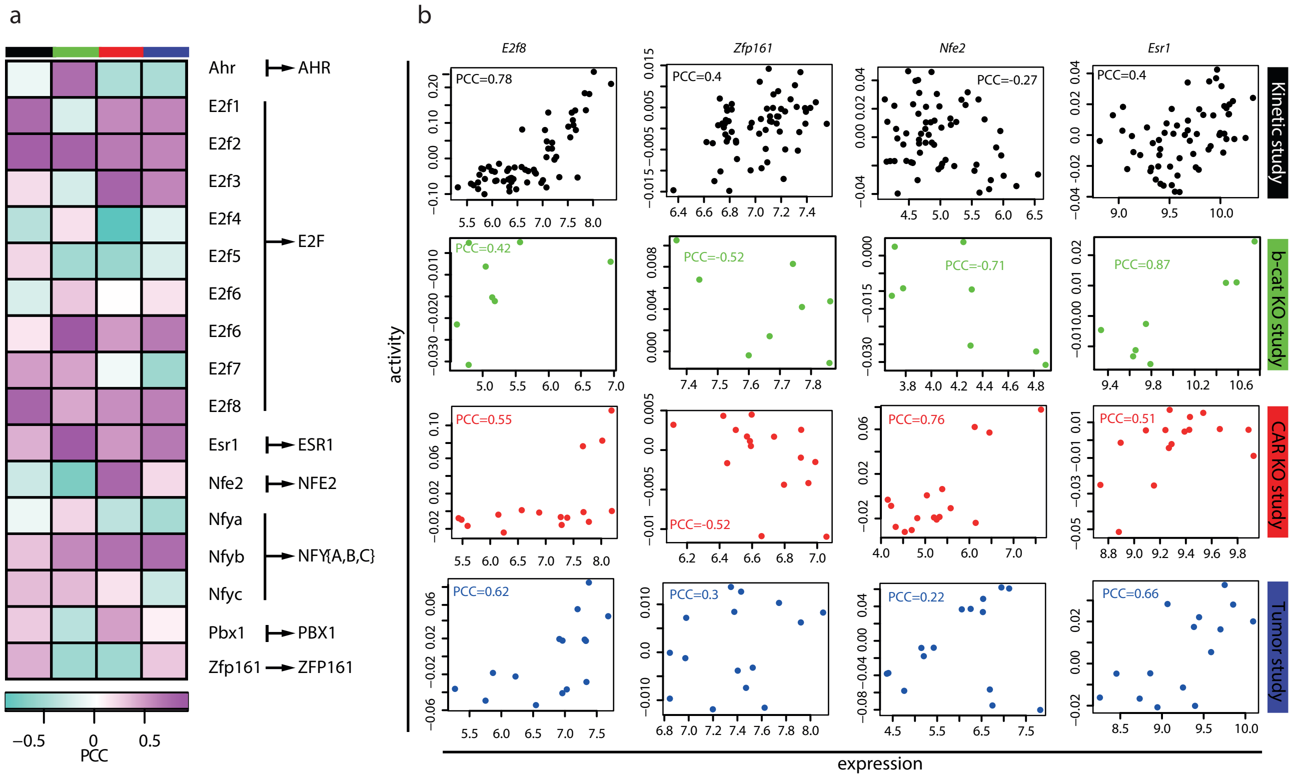

Figure S3: Correlation between motif activities and mRNA expression of cognate transcription factors. **(a)** Heatmap of the Pearson correlation coefficients (PCC) between the motif activities and mRNA expression profiles of associated TFs for a selection of TFs specifically dysregulated in promoted tumors. Each column corresponds to one of the 4 experimental data-sets (black = kinetic study, green = β-catenin KO study, red = CAR KO study and blue = tumor study) and PCC is indicated by color running from -1 (green), to 1 (purple). PCCs close to zero are colored white. **(b)** Scatter plots of motif activities against mRNA expression of associated TFs for a selection of 4 TFs. Each column of panels corresponds to one TF and each row of panels corresponds to one of the 4 experimental data-sets.

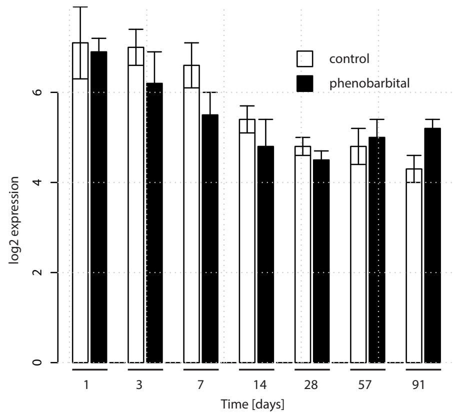

Figure S4: Alpha fetoprotein (Afp) gene expression in liver samples from 13 week kinetic data-sets as a surrogate gene of post-natal liver development termination. Gene expression is given as mean ±SD (n=3-5 animals per group). Open bars = control. Black bars = phenobarbital-treated samples.

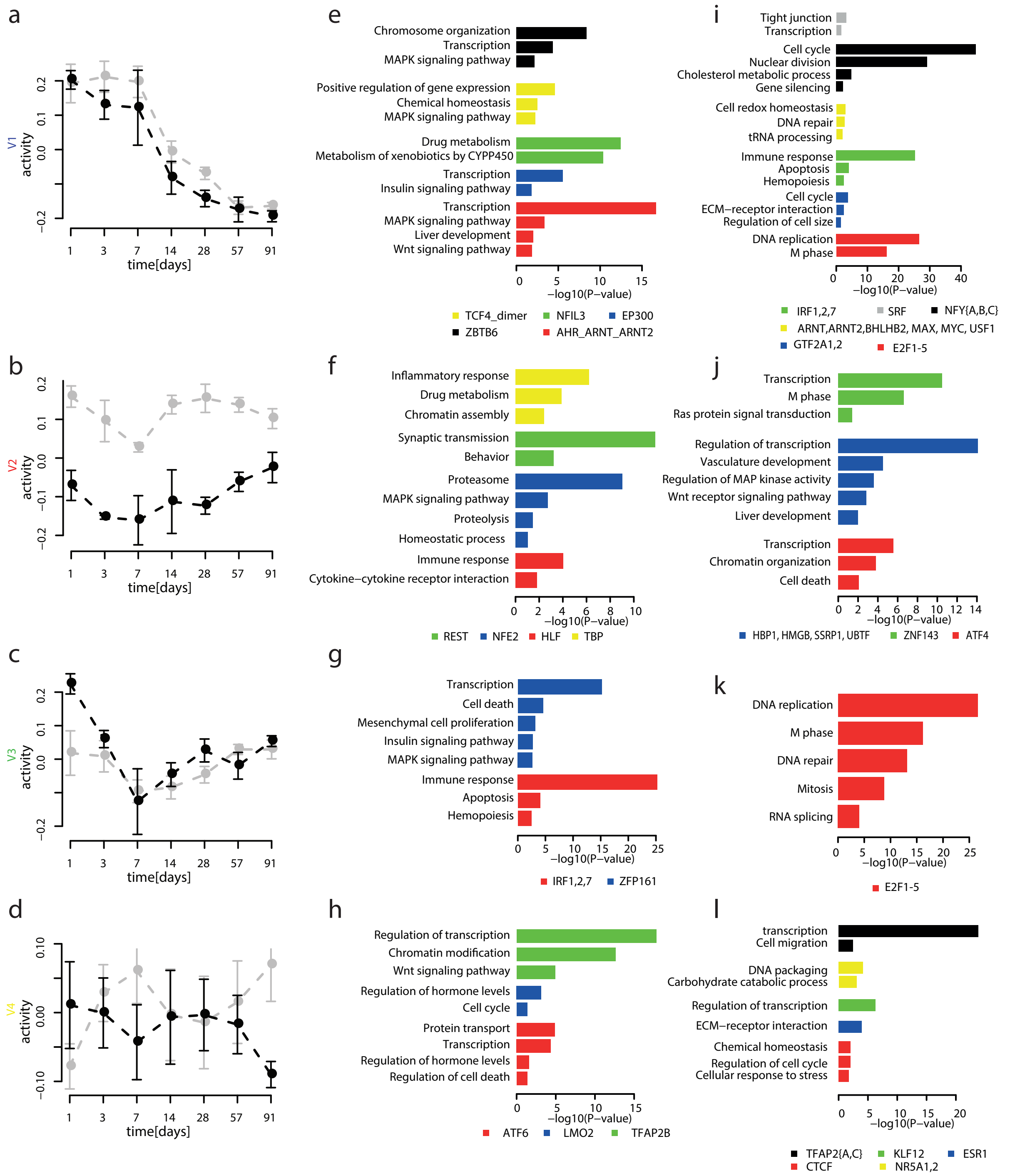

Figure S5: Gene Ontology and KEGG enrichment analysis of predicted targets for motifs underlying early PB-mediated transcriptional dynamics. **(a-d)** Plots of the activity profiles of the first four right singular vectors. **(e)-(l)** Selection of biological pathways and functional categories (Gene Ontology or KEGG) enriched among target genes of motifs that contribute/correlate negatively **(e-h)** or positively **(i-l)** to each of the singular vectors. Each color corresponds to one regulatory motif, indicated at the bottom of each panel, and the size of each bar corresponds to the significance ($-\log_{10}(p-\text{value})$) of the enrichment.

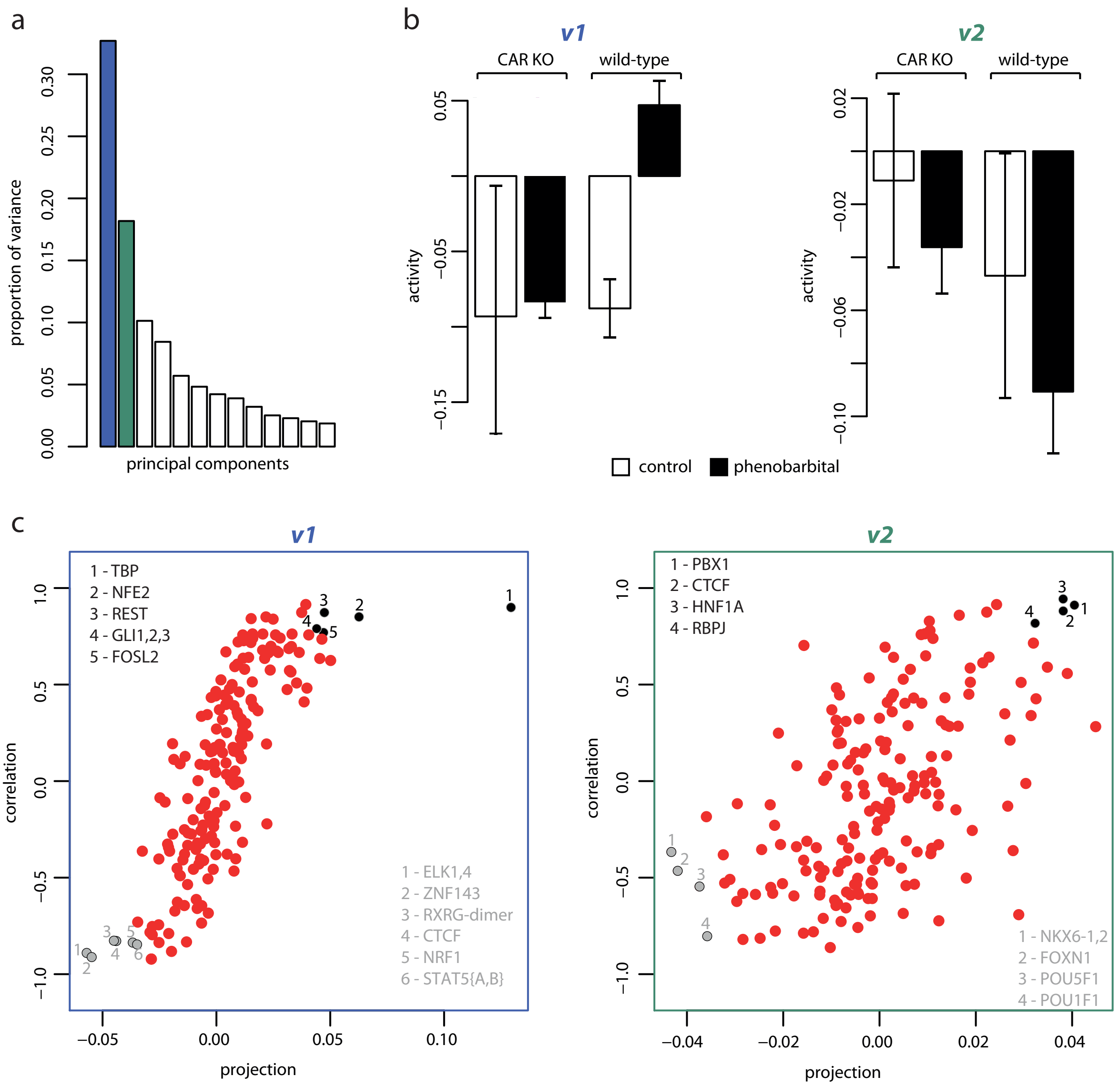

Figure S6: Singular Value Decomposition analysis of the activity matrix of the CAR KO data-set. **(a)** Proportion of the variance of the motif activity matrix. The first (blue bar) and second (green bar) components account for 33% and 18% respectively of the variance. **(b)** Barplot of the activity of the first two right singular vectors $v1$ and $v2$ in corresponding samples. White bars indicate activities for the control samples and black bars activities for the PB-treated samples. **(c)** Scatter plot of the correlations ρ_i and projections p_i of all motifs i with the first and second right singular vectors respectively. Grey and black dots depict negatively and positively selected motifs.

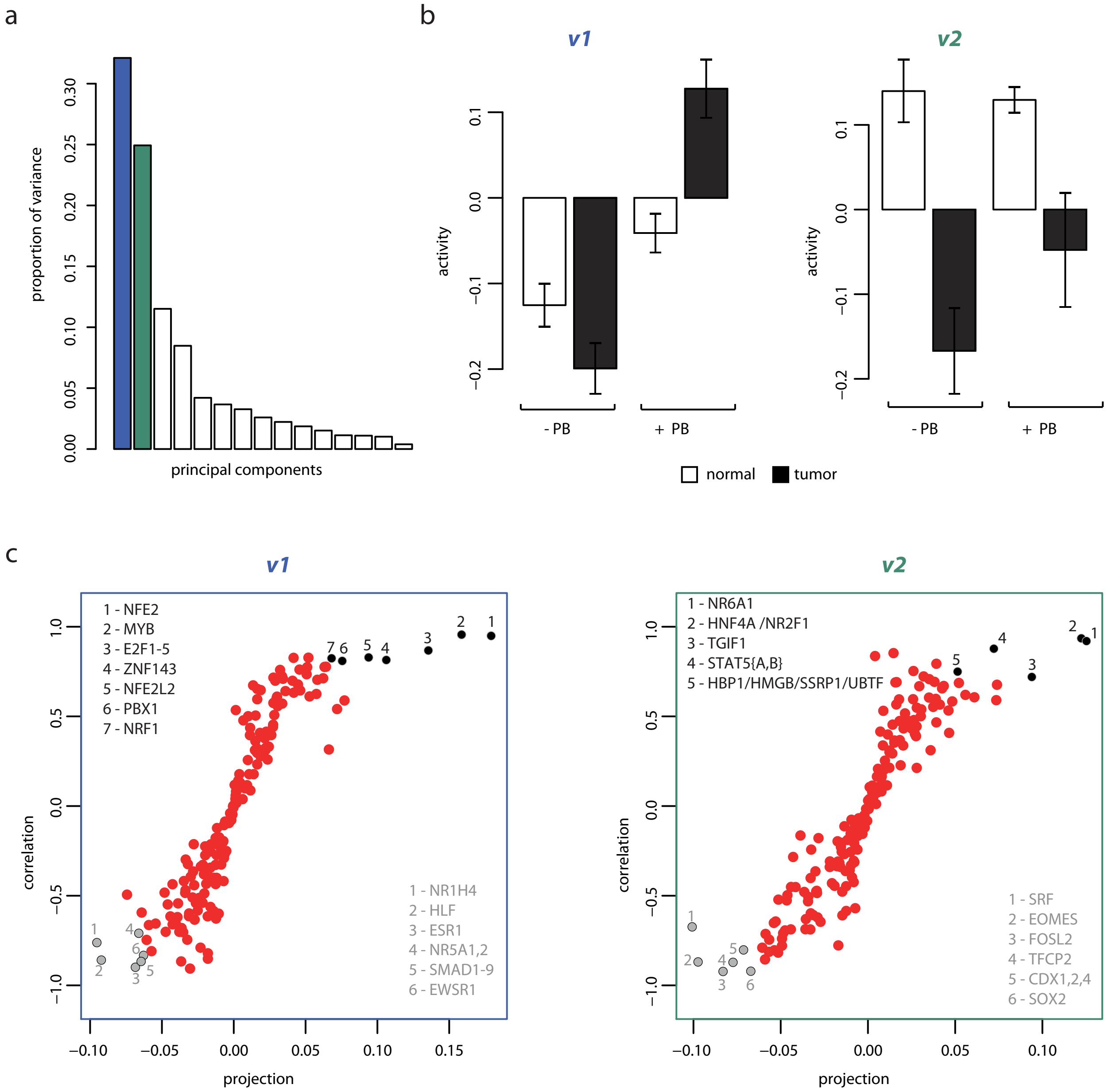

Figure S7: Singular Value Decomposition analysis of the activity matrix of the tumor data-set. **(a)** Proportion of the variance of the motif activity matrix captured by the first singular vectors. The first (blue bar) and second (green bar) components account for 32% and 25% respectively of the variance. **(b)** Barplot of the activity of the first two right singular vectors $v1$ and $v2$ across the corresponding samples. White bars indicate activities for the normal samples and black bars activities for the tumor samples. **(c)** Scatter plot of the correlations ρ_i and projections p_i of all motifs i with the first and second right singular vectors respectively. Grey and black dots depict negatively and positively selected motifs.

4 Abbreviations contained in Tables S1-S5

Tables S1-S5 contain motifs corresponding to specific groups that are

1. Table S1 - motifs associated with the first four singular vectors obtained from singular value decomposition (SVD) of the inferred motifs activity matrix from early kinetic study
2. Table S2 - motifs down-stream of CAR signaling
3. Table S3 - motifs dysregulated in both promoted and non-promoted tumors
4. Table S4 - motifs specifically dysregulated in promoted tumors
5. Table S5 - motifs down-stream of β-catenin signaling.

They are all formatted in the same way and their abbreviations are described in the following:

1. Representative motifs associated with the first four singular vectors obtained from SVD of the inferred motifs activity matrix from early kinetic study

   PC1 = first singular vector associated with liver maturation

   PC2 = second singular vector associated with constant xenobiotic response

   PC3 = third singular vector associated with transient mitogenic response

   PC4 = fourth singular vector associated with progressive xenobiotic response

   $+$ = motifs correlating positively with corresponding singular vector

   $-$ = motifs correlating negatively with corresponding singular vector

2. Z-value of motif significance that quantifies the significance of each motif in explaining the observed gene expression variation across the samples in the specified data-set

   S1 = kinetic data-set

   S2 = β-catenin KO data-set

   S3 = CAR KO data-set

   S4 = tumor data-set.

3. Z-values of differential motif activity that quantifies the evidence for a different regulatory activity of the motif between the tow following conditions

   $\mathbf{d}_i$ = PB-treated and control samples at corresponding time-point

   KO = knock-out and wild-type samples

   $\mathbf{PB}, wt$ = PB-treated and non-treated wild-type samples of the KO data-sets

   $\mathbf{PB}, ko$ = PB-treated and non-treated KO samples of the KO data-sets

   β**-catenin** = promoted tumors and treated surrounding normal tissue

   H-ras = non-promoted tumors and surrounding non-treated normal tissue.

5 Supplementary Tables

	Representative motifs				Motif Significance [z-value]				Differential Motif Activity												
									Kinetic study							b-catenin study	CAR study			Tumor study	
	PC1	PC2	PC3	PC4	S1	S2	S3	S4	d1	d3	d7	d14	d28	d57	d91	KO	KO	PB, *wt*	PB, *ko*	β-catenin	H-ras
E2F	+		+		3.7	1.8	2.3	2.7	**2.2**	**-0.7**	**-0.5**	**-1.2**	**-1.3**	**-0.8**	**0.3**	-2.5	-0.6	-0.7	-0.1	2.6	-0.3
IRF1,2,7	+		-		5.4	3.3	1.7	2.0	**-1.8**	**-0.8**	**0.2**	**-0.5**	**-1.7**	**0.6**	**-1.3**	3.7	0.6	1.8	0.3	0.6	0.1
SRF	+				3.0	1.3	0.7	1.9	**-2.4**	**-1.0**	**-1.0**	**-2.2**	**-0.6**	**0.4**	**-0.1**	1.7	0.2	-0.5	-1.0	1.0	1.9
NFY{A,B,C}	+				2.9	0.7	2.2	1.0	**1.3**	**-2.8**	**-3.0**	**-0.4**	**-0.3**	**-1.6**	**-1.3**	-1.0	0.2	-1.7	-0.2	1.7	0.8
GTF2A1,2	+				2.2	1.7	0.8	0.5	**3.1**	**1.2**	**-0.3**	**0.7**	**1.7**	**1.5**	**-0.5**	2.1	0.5	0.4	-0.5	-0.3	0.2
ARNT,ARNT2,BHLHB2,MAX,MYC,USF1	+				2.8	1.2	0.7	1.0	**2.2**	**-0.2**	**-2.4**	**-0.4**	**0.3**	**0.0**	**-0.8**	1.7	-0.9	-1.0	0.2	0.4	2.1
ZBTB6	-				2.2	0.4	1.1	1.3	**-1.8**	**-0.7**	**-0.1**	**-0.3**	**-0.2**	**-0.9**	**1.4**	-0.4	0.1	1.5	-0.5	-2.4	-1.5
TCF4-dimer	-				2.5	1.2	1.3	1.9	**0.1**	**0.4**	**0.5**	**0.0**	**-0.1**	**-2.1**	**-1.9**	-1.3	-0.1	-0.2	-0.2	-1.9	-3.2
NFIL3	-				2.4	1.5	1.3	0.9	**0.9**	**2.8**	**3.2**	**2.5**	**3.6**	**0.3**	**3.2**	-2.2	-0.9	0.9	0.7	0.6	-0.6
EP300	-				1.9	2.8	0.8	1.4	**0.1**	**0.4**	**0.9**	**0.1**	**0.1**	**0.4**	**-1.2**	-3.9	0.6	0.2	-0.1	-0.4	-2.3
AHR,ARNT,ARNT2	-				2.4	0.1	1.5	1.6	**-1.4**	**0.0**	**0.8**	**-0.6**	**-0.4**	**-2.0**	**-0.1**	0.2	0.1	-2.1	0.2	-1.9	-1.2
ZNF143		+			1.8	1.0	2.1	1.7	**0.0**	**-2.5**	**-2.3**	**-2.6**	**-2.1**	**-2.6**	**-2.1**	-1.3	-0.2	-3.0	-0.2	0.4	-0.1
HBP1,HMGB,SSRP1,UBTF		+			2.9	1.2	1.2	1.5	**-6.0**	**-4.1**	**-1.9**	**-2.4**	**-5.1**	**-2.6**	**-1.8**	1.7	0.9	0.7	0.8	-2.2	-0.9
ATF4		+			1.6	1.1	0.7	0.8	**-2.8**	**-2.4**	**-1.0**	**-1.5**	**-1.6**	**-3.3**	**-0.6**	1.5	0.1	-1.0	-0.5	0.6	0.8
TBP		-			2.7	0.3	2.2	0.5	**4.6**	**2.7**	**2.3**	**2.1**	**3.0**	**5.2**	**2.5**	0.3	-0.6	2.5	-0.4	-0.1	-0.5
REST		-			2.4	0.6	1.5	0.8	**2.3**	**2.8**	**4.4**	**2.0**	**4.0**	**1.8**	**0.7**	-0.5	0.0	1.8	0.1	0.5	-0.2
NFE2		-			1.7	1.6	3.0	2.6	**3.9**	**1.4**	**1.6**	**1.4**	**2.6**	**0.8**	**-0.4**	-2.2	-0.3	2.3	1.0	2.0	-1.4
HLF		-			1.8	0.5	1.3	1.4	**1.4**	**2.3**	**1.2**	**3.0**	**2.5**	**1.3**	**3.2**	0.7	0.4	2.6	-0.2	-1.4	1.1
ZFP161			-		1.6	0.3	1.0	1.6	**-2.0**	**-1.0**	**0.0**	**-1.1**	**-1.3**	**-0.7**	**1.9**	-0.2	-0.6	0.1	0.6	-1.6	0.7
TFAP2{A,C}				+	2.2	0.7	2.8	2.7	**-1.8**	**-1.9**	**-1.9**	**-1.1**	**-1.3**	**-1.4**	**-4.0**	1.0	0.8	-1.1	-0.5	-1.6	-3.7
NR5A1,2				+	1.2	2.0	2.1	2.3	**0.8**	**-0.1**	**0.8**	**0.4**	**0.4**	**0.5**	**-2.6**	1.9	1.7	-0.5	-1.8	-2.8	-2.0
KLF12				+	1.7	0.7	1.6	1.4	**0.4**	**-0.3**	**-1.4**	**0.6**	**0.8**	**0.8**	**-1.9**	1.0	0.2	-1.4	-0.2	-2.2	0.0
ESR1				+	1.7	1.9	1.5	1.9	**0.6**	**-0.7**	**-1.4**	**-0.1**	**-0.7**	**-0.8**	**-2.7**	2.6	1.4	0.0	-0.3	-2.4	0.5
CTCF				+	1.6	0.2	0.7	1.2	**-0.2**	**-2.3**	**-4.6**	**-1.5**	**-1.8**	**-1.1**	**-2.1**	0.3	0.7	-1.1	-0.5	0.6	2.8
TFAP2B				-	2.0	2.1	0.7	1.4	**-1.7**	**-1.1**	**0.5**	**-0.1**	**-0.5**	**-1.0**	**2.4**	-2.6	-0.7	-0.6	0.0	0.2	-0.6
LMO2				-	1.2	0.4	1.8	1.3	**-0.8**	**-0.3**	**-0.3**	**-0.3**	**0.3**	**2.6**	**2.0**	-0.4	-0.9	1.8	0.1	1.4	1.5
ATF6				-	1.1	0.6	0.7	1.4	**-1.8**	**-0.1**	**0.0**	**-0.2**	**0.6**	**0.8**	**2.5**	-0.9	0.0	0.7	-0.3	2.1	1.9

Table S1: Representative motifs of the first four singular vectors (explaining over 70% of the variance in the activity matrix) obtained from singular value decomposition of the inferred motifs activity matrix from early kinetic study, and underlying the early dysregulated biological pathways. Z-values of differential activity were computed as explained in Material and Method section of the main manuscript.

	Representative motifs				Motif Significance [z-value]				Differential Motif Activity												
									Kinetic study							b-catenin study	CAR study			Tumor study	
	PC1	PC2	PC3	PC4	S1	S2	S3	S4	d1	d3	d7	d14	d28	d57	d91	KO	KO	PB, *wt*	PB, *ko*	β-catenin	H-ras
**Motifs down-stream of CAR signaling under physiological condition**																					
NKX3-2					1.5	0.2	2.6	1.5	1.5	-0.3	-1.1	0.5	1.8	1.1	0.0	0.2	**2.7**	2.1	-1.3	1.7	2.6
FOX{F1,F2,J1}					1.6	0.2	1.9	1.1	0.8	2.0	1.2	1.3	0.5	2.4	0.8	0.2	**1.8**	2.2	-0.7	0.3	-0.4
NR5A1,2				+	1.2	2.0	2.1	2.3	0.8	-0.1	0.8	0.4	0.4	0.5	-2.6	1.9	**1.7**	-0.5	-1.8	-2.8	-2.0
ONECUT1,2					2.0	1.0	1.5	0.8	-1.5	-2.2	-2.4	-2.1	-1.3	-1.5	-1.6	-1.4	**-1.6**	-0.5	0.0	0.2	0.6
NKX2-2,8					1.5	1.2	2.2	1.4	-2.0	-0.9	0.9	-0.7	-0.8	0.4	1.7	1.7	**-1.6**	2.1	0.4	-0.7	-1.0
**Motifs down-stream of CAR signaling under PB treatment**																					
FOX{I1,J2}					1.6	0.2	2.2	0.4	0.5	1.7	1.6	1.2	1.3	2.9	0.7	0.3	1.1	**3.8**	**1.0**	-0.2	0.3
NFKB1,REL,RELA					1.8	0.7	2.2	2.1	0.5	1.9	-0.3	1.0	1.0	0.9	1.0	-0.5	-0.7	**2.7**	**0.3**	0.2	0.9
TBP		-			2.7	0.3	2.2	0.5	4.6	2.7	2.3	2.1	3.0	5.2	2.5	0.3	-0.6	**2.5**	**-0.4**	-0.1	-0.5
NFE2		-			1.7	1.6	3.0	2.6	3.9	1.4	1.6	1.4	2.6	0.8	-0.4	-2.2	-0.3	**2.3**	**1.0**	2.0	-1.4
PRDM1					1.4	0.5	2.2	1.1	0.0	1.1	0.7	0.9	2.1	0.9	0.8	0.7	0.8	**2.2**	**-0.2**	1.7	-0.2
FOX{F1,F2,J1}					1.6	0.2	1.9	1.1	0.8	2.0	1.2	1.3	0.5	2.4	0.8	0.2	1.8	**2.2**	**-0.7**	0.3	-0.4
NKX3-2					1.5	0.2	2.6	1.5	1.5	-0.3	-1.1	0.5	1.8	1.1	0.0	0.2	2.7	**2.1**	**-1.3**	1.7	2.6
NKX2-2,8					1.5	1.2	2.2	1.4	-2.0	-0.9	0.9	-0.7	-0.8	0.4	1.7	1.7	-1.6	**2.1**	**0.4**	-0.7	-1.0
REST		-			2.4	0.6	1.5	0.8	2.3	2.8	4.4	2.0	4.0	1.8	0.7	-0.5	0.0	**1.8**	**0.1**	0.5	-0.2
IRF1,2,7	+		-		5.4	3.3	1.7	2.0	-1.8	-0.8	0.2	-0.5	-1.7	0.6	-1.3	3.7	0.6	**1.8**	**0.3**	0.6	0.1
LMO2				-	1.2	0.4	1.8	1.3	-0.8	-0.3	-0.3	-0.3	0.3	2.6	2.0	-0.4	-0.9	**1.8**	**0.1**	1.4	1.5
FOSL2					0.7	0.7	1.6	1.4	-0.3	-0.4	0.0	0.4	-0.2	-0.1	1.1	0.9	0.1	**1.7**	**0.1**	1.2	2.4
RXR{A,B,G}					1.7	2.5	1.5	1.1	1.9	2.1	0.8	1.3	1.9	0.9	-0.6	3.5	0.0	**1.6**	**0.4**	-1.0	1.0
HOX{A4,D4}					2.2	0.7	1.7	1.1	0.1	0.5	0.8	1.3	-0.2	2.2	-0.4	0.8	1.2	**1.6**	**0.4**	-2.0	-0.7
GLI1-3					0.9	1.3	1.9	1.0	-1.5	-0.5	-0.9	-1.0	0.1	-0.5	-1.7	1.8	0.0	**1.5**	**-0.2**	0.4	-0.2
NFY{A,B,C}	+				2.9	0.7	2.2	1.0	1.3	-2.8	-3.0	-0.4	-0.3	-1.6	-1.3	-1.0	0.2	**-1.7**	**-0.2**	1.7	0.8
AHR,ARNT,ARNT2	-				2.4	0.1	1.5	1.6	-1.4	0.0	0.8	-0.6	-0.4	-2.0	-0.1	0.2	0.1	**-2.1**	**0.2**	-1.9	-1.2
CREB1					1.3	0.1	2.0	1.1	-0.2	-0.7	-1.7	-1.3	-1.2	-1.0	-0.4	-0.2	-0.6	**-2.1**	**0.6**	1.4	1.7
ELF1,2,4					2.2	0.5	1.9	2.6	-4.2	-2.7	-2.9	-2.5	-3.8	-1.8	-2.0	0.4	0.2	**-2.2**	**-0.3**	1.7	5.7
ELK1,4,GABP{A,B1}					2.3	0.6	1.9	1.1	0.1	-2.3	-1.4	-1.6	-0.8	-1.5	-1.2	-0.8	-0.1	**-2.6**	**-0.4**	0.8	1.3
ZNF143		+			1.8	1.0	2.1	1.7	0.0	-2.5	-2.3	-2.6	-2.1	-2.6	-2.1	-1.3	-0.2	**-3.0**	**-0.2**	0.4	-0.1
NRF1					1.5	0.5	1.8	1.8	1.1	-1.8	-1.3	-0.7	0.0	-0.6	-1.0	-0.6	-0.2	**-3.1**	**-0.3**	0.4	0.3
FOXD3					2.3	1.3	1.9	1.4	-3.0	-3.0	-1.5	-2.5	-4.1	-1.3	-0.9	0.9	0.0	**-3.6**	**-0.9**	-1.8	-0.7
**Motifs differentially active upon PB treatment only in KO**																					
SPI1					1.3	3.1	1.5	1.3	-3.9	-0.6	0.1	-0.5	-0.4	-2.0	1.2	4.3	-0.5	**-0.6**	**1.7**	-0.4	1.2
ZNF148					1.9	1.2	1.6	0.7	3.6	1.3	0.1	0.0	0.1	-0.2	-2.0	-1.7	0.8	**0.1**	**-2.0**	-0.1	-0.7
NR5A1,2				+	1.2	2.0	2.1	2.3	0.8	-0.1	0.8	0.4	0.4	0.5	-2.6	1.9	1.7	**-0.5**	**-1.8**	-2.8	-2.0
HNF4A,NR2F1,2					0.9	1.8	1.6	2.2	0.5	-0.4	-1.2	0.2	0.6	-0.2	-0.9	-2.2	0.2	**0.3**	**-1.6**	-1.5	-2.1

Table S2: Motifs which activities are significantly changing either 1) upon CAR KO in non-treated samples and thus potentially down-stream of CAR signaling under physiological condition, or under PB treatment 2) only in CAR wild-type samples and thus potentially down-stream of CAR signaling under PB treatment, or 3) only in CAR KO samples. Z-values of differential activity were computed as explained in Material and Method section of the main manuscript.

	Representative motifs				Motif Significance [z-value]				Differential Motif Activity												
									Kinetic study							b-catenin study	CAR study			Tumor study	
	**PC1**	**PC2**	**PC3**	**PC4**	**S1**	**S2**	**S3**	**S4**	**d1**	**d3**	**d7**	**d14**	**d28**	**d57**	**d91**	**KO**	**KO**	**PB**, *wt*	**PB**, *ko*	**β-catenin**	**H-ras**
TFAP2{A,C}				+	2.2	0.7	2.8	2.7	-1.8	-1.9	-1.9	-1.1	-1.3	-1.4	-4.0	1.0	0.8	-1.1	-0.5	**-1.6**	**-3.7**
NR6A1					1.4	0.8	1.0	2.0	-0.4	0.4	0.9	-1.2	-1.0	-0.1	-2.0	-1.2	0.3	-1.0	-0.7	**-2.0**	**-3.5**
TCF4-dimer	-				2.5	1.2	1.3	1.9	0.1	0.4	0.5	0.0	-0.1	-2.1	-1.9	-1.3	-0.1	-0.2	-0.2	**-1.9**	**-3.2**
GATA6					0.9	0.8	1.6	1.7	-0.5	0.2	0.9	-0.4	-0.1	-1.4	-0.3	-0.9	0.5	0.1	-0.5	**-2.7**	**-2.3**
NR5A1,2				+	1.2	2.0	2.1	2.3	0.8	-0.1	0.8	0.4	0.4	0.5	-2.6	1.9	1.7	-0.5	-1.8	**-2.8**	**-2.0**
NR1H4					1.7	0.1	1.8	2.8	-1.4	-1.4	-0.4	-0.2	-0.2	-0.1	-1.6	0.0	0.9	0.1	-0.8	**-4.7**	**-1.7**
SOX{8,9,10}					1.0	0.6	1.0	1.6	-1.0	-0.7	0.1	-0.1	-1.2	0.7	-1.8	0.6	-0.8	-0.2	0.6	**2.0**	**2.5**

Table S3: Motifs which activities are significantly changing in promoted tumors relative to surrounding treated normal tissue, and in non-promoted tumors relative to surrounding non-treated normal tissue. These motifs are thus candidate regulators of liver tumorigenesis. Z-values of differential activity were computed as explained in Material and Method section of the main manuscript.

	Representative motifs				Motif Significance [z-value]				Differential Motif Activity													
									Kinetic study							b-catenin study	CAR study			Tumor study	
	**PC1**	**PC2**	**PC3**	**PC4**	**S1**	**S2**	**S3**	**S4**	**d1**	**d3**	**d7**	**d14**	**d28**	**d57**	**d91**	**KO**	**KO**	**PB**, *wt*	**PB**, *ko*	β**-catenin**	**H-ras**
NFE2		-			1.7	1.6	3.0	2.6	3.9	1.4	1.6	1.4	2.6	0.8	-0.4	-2.2	-0.3	2.3	1.0	**2.0**	**-1.4**
AHR,ARNT,ARNT2	-				2.4	0.1	1.5	1.6	-1.4	0.0	0.8	-0.6	-0.4	-2.0	-0.1	0.2	0.1	-2.1	0.2	**-1.9**	**-1.2**
E2F	+		+		3.7	1.8	2.3	2.7	2.2	-0.7	-0.5	-1.2	-1.3	-0.8	0.3	-2.5	-0.6	-0.7	-0.1	**2.6**	**-0.3**
ESR1				+	1.7	1.9	1.5	1.9	0.6	-0.7	-1.4	-0.1	-0.7	-0.8	-2.7	2.6	1.4	0.0	-0.3	**-2.4**	**0.5**
ZFP161			-		1.6	0.3	1.0	1.6	-2.0	-1.0	0.0	-1.1	-1.3	-0.7	1.9	-0.2	-0.6	0.1	0.6	**-1.6**	**0.7**
PBX1					1.3	0.4	1.8	1.8	1.2	-0.1	0.6	-0.7	0.0	0.8	-0.6	-0.5	0.9	-0.4	-0.8	**3.1**	**0.8**
NFY{A,B,C}	+				2.9	0.7	2.2	1.0	1.3	-2.8	-3.0	-0.4	-0.3	-1.6	-1.3	-1.0	0.2	-1.7	-0.2	**1.7**	**0.8**

Table S4: Motifs which activities are significantly changing in promoted tumors relative to surrounding treated normal tissue, but not in non-promoted tumors relative to surrounding non-treated normal tissue. These motifs are thus candidate regulators of tumor promotion. Z-values of differential activity were computed as explained in Material and Method section of the main manuscript.

	Representative motifs				Motif Significance [z-value]				Differential Motif Activity												
									Kinetic study							b-catenin study	CAR study			Tumor study	
	**PC1**	**PC2**	**PC3**	**PC4**	**S1**	**S2**	**S3**	**S4**	**d1**	**d3**	**d7**	**d14**	**d28**	**d57**	**d91**	**KO**	**KO**	**PB**, *wt*	**PB**, *ko*	β**-catenin**	**H-ras**
HNF1A					1.3	3.7	1.0	0.6	-2.7	-2.1	-0.9	-0.6	-1.8	-1.6	-0.9	**-4.7**	0.7	-1.0	-0.5	0.0	0.7
EP300	-				1.9	2.8	0.8	1.4	0.1	0.4	0.9	0.1	0.1	0.4	-1.2	**-3.9**	0.6	0.2	-0.1	-0.4	-2.3
POU6F1					0.8	2.2	0.7	0.6	-1.2	-0.8	-0.3	-1.7	-1.5	-0.6	0.2	**-2.9**	0.3	-0.3	0.8	-0.2	-0.5
LEF1,TCF7,TCF7L1,2					1.2	2.0	0.5	0.7	0.3	-0.3	1.2	-0.7	-1.4	0.2	0.0	**-2.8**	-0.1	-0.3	0.5	1.0	-0.6
TFAP2B				-	2.0	2.1	0.7	1.4	-1.7	-1.1	0.5	-0.1	-0.5	-1.0	2.4	**-2.6**	-0.7	-0.6	0.0	0.2	-0.6
ZNF384					2.0	1.8	0.5	1.0	-3.6	-2.0	-1.9	-2.1	-3.0	-1.9	0.8	**-2.6**	-0.9	-0.7	-0.1	1.3	-0.8
CDX1,2,4					3.0	1.9	1.2	2.5	2.1	2.8	0.1	2.3	2.3	-0.7	2.0	**-2.5**	-1.1	-0.6	0.1	4.0	0.9
E2F	+		+		3.7	1.8	2.3	2.7	2.2	-0.7	-0.5	-1.2	-1.3	-0.8	0.3	**-2.5**	-0.6	-0.7	-0.1	2.6	-0.3
NFE2L2					1.3	1.9	0.4	1.9	1.9	0.7	1.8	2.1	1.0	1.5	1.6	**-2.5**	0.0	0.7	-0.2	0.7	-2.2
RFX1-5,RFXANK,RFXAP					1.5	2.7	1.3	1.2	1.4	1.5	-1.0	1.1	0.7	0.6	-0.5	**-2.4**	-0.2	-0.3	-0.2	0.7	0.3
NFE2		-			1.7	1.6	3.0	2.6	3.9	1.4	1.6	1.4	2.6	0.8	-0.4	**-2.2**	-0.3	2.3	1.0	2.0	-1.4
ATF5,CREB3					1.2	2.0	0.4	0.6	-0.9	-1.2	0.1	-0.1	0.2	0.4	1.1	**-2.2**	0.2	-0.5	-0.6	-0.7	-0.3
HNF4A,NR2F1,2					0.9	1.8	1.6	2.2	0.5	-0.4	-1.2	0.2	0.6	-0.2	-0.9	**-2.2**	0.2	0.3	-1.6	-1.5	-2.1
NFIL3	-				2.4	1.5	1.3	0.9	0.9	2.8	3.2	2.5	3.6	0.3	3.2	**-2.2**	-0.9	0.9	0.7	0.6	-0.6
POU5F1,SOX2{dimer}					2.1	1.9	0.9	0.7	1.8	3.3	0.0	2.2	3.7	0.2	1.3	**-1.8**	-0.7	0.0	0.7	-0.3	-0.6
MYB					1.6	1.8	2.2	2.7	0.4	-0.3	0.3	-0.9	-0.5	-1.0	0.8	**-1.5**	-1.0	-1.1	-0.1	4.2	-0.4
GATA1-3					1.6	1.5	1.3	0.7	1.7	0.3	-2.5	1.1	0.9	0.8	-0.5	**1.7**	-0.3	1.5	1.6	-0.1	-0.2
FOXL1					1.9	1.6	1.6	1.0	-2.1	-1.7	-1.1	-3.1	-3.4	-1.6	-0.3	**1.8**	-0.1	-1.1	0.3	1.6	1.3
NR5A1,2				+	1.2	2.0	2.1	2.3	0.8	-0.1	0.8	0.4	0.4	0.5	-2.6	**1.9**	1.7	-0.5	-1.8	-2.8	-2.0
PAX2					1.8	1.6	1.0	0.7	-1.6	-3.0	-3.1	-3.0	-1.8	-2.0	-1.2	**2.0**	-0.1	0.2	-0.4	0.5	0.8
GTF2A1,2	+				2.2	1.7	0.8	0.5	3.1	1.2	-0.3	0.7	1.7	1.5	-0.5	**2.1**	0.5	0.4	-0.5	-0.3	0.2
TFAP4					1.5	2.0	1.2	0.5	-0.8	0.0	0.5	0.0	0.4	0.6	0.4	**2.3**	0.5	2.3	-0.3	-0.7	-0.1
MTF1					2.1	2.0	1.2	1.1	1.5	2.7	-0.3	0.5	1.1	2.0	-1.2	**2.6**	0.2	2.1	0.5	-0.7	-2.3
ESR1				+	1.7	1.9	1.5	1.9	0.6	-0.7	-1.4	-0.1	-0.7	-0.8	-2.7	**2.6**	1.4	0.0	-0.3	-2.4	0.5
KLF4					2.2	2.7	0.7	1.2	1.0	0.8	-1.4	-0.1	0.0	0.2	-1.4	**2.8**	0.5	-0.2	-0.1	1.1	-0.2
TFCP2					1.5	2.7	1.1	2.6	0.1	-1.7	-1.3	-0.2	-0.3	-0.1	0.2	**3.3**	0.8	0.2	0.5	0.7	3.7
RUNX1-3					1.1	2.6	1.2	0.9	1.3	-0.1	0.5	0.9	1.1	1.1	-0.4	**3.3**	1.0	1.5	-0.5	-0.8	-0.1
RXR{A,B,G}					1.7	2.5	1.5	1.1	1.9	2.1	0.8	1.3	1.9	0.9	-0.6	**3.5**	0.0	1.6	0.4	-1.0	1.0
IRF1,2,7	+		-		5.4	3.3	1.7	2.0	-1.8	-0.8	0.2	-0.5	-1.7	0.6	-1.3	**3.7**	0.6	1.8	0.3	0.6	0.1
TEAD1					1.0	2.9	1.0	0.9	-0.1	0.4	0.0	-0.3	-0.3	1.0	0.4	**4.1**	0.4	1.1	0.1	-0.3	2.0
FEV					2.5	3.0	0.6	0.8	1.4	0.7	-0.2	0.0	0.0	-0.1	-1.5	**4.3**	0.5	1.2	0.0	-0.1	-0.1
SPI1					1.3	3.1	1.5	1.3	-3.9	-0.6	0.1	-0.5	-0.4	-2.0	1.2	**4.3**	-0.5	-0.6	1.7	-0.4	1.2
FOS,FOS{B,L1},JUN{B,D}					1.5	3.4	0.4	1.5	-2.4	-1.9	-0.7	-1.8	-2.1	-1.3	-1.3	**4.8**	-0.3	0.1	0.7	0.2	2.3

Table S5: Motifs which activities are significantly changing upon β-catenin KO in non-treated samples and thus potentially down-stream of β-catenin signaling under physiological condition. Z-values of differential activity were computed as explained in Material and Method section of the main manuscript.

Affx	GS	Motifs	Kinetic study PCC	Kinetic study P-value	β-catenin study PCC	β-catenin study P-value	CAR KO study PCC	CAR KO study P-value	Tumor Study PCC	Tumor Study P-value
1450695_at	Ahr	AHR,ARNT,ARNT2	-0.09	5.1E-01	0.73	3.87E-02	-0.43	9.3E-02	-0.45	9.2E-02
1421721_a_at	Arnt	ARNT,ARNT2,BHLHB2,MAX,MYC,USF1	0.20	1.1E-01	-0.45	2.62E-01	0.43	9.9E-02	0.06	8.4E-01
1434028_at	Arnt2	ARNT,ARNT2,BHLHB2,MAX,MYC,USF1	0.07	5.9E-01	-0.19	6.54E-01	-0.05	8.6E-01	0.63	1.2E-02
1418025_at	Bhlhe40	ARNT,ARNT2,BHLHB2,MAX,MYC,USF1	-0.41	8.1E-04	0.26	5.26E-01	0.33	2.1E-01	0.33	2.3E-01
1423501_at	Max	ARNT,ARNT2,BHLHB2,MAX,MYC,USF1	0.01	9.3E-01	-0.07	8.65E-01	0.04	8.8E-01	-0.06	8.3E-01
1424942_a_at	Myc	ARNT,ARNT2,BHLHB2,MAX,MYC,USF1	0.11	4.1E-01	-0.20	6.36E-01	-0.02	9.5E-01	-0.28	3.2E-01
1448805_at	Usf1	ARNT,ARNT2,BHLHB2,MAX,MYC,USF1	0.10	4.2E-01	0.55	1.54E-01	0.25	3.4E-01	0.34	2.1E-01
1438992_x_at	Atf4	ATF4	0.21	1.0E-01	-0.23	5.76E-01	0.42	1.1E-01	-0.54	3.8E-02
1425927_a_at	Atf5	ATF5,CREB3	-0.40	1.1E-03	0.70	5.15E-02	0.28	2.9E-01	-0.51	5.3E-02
1419979_s_at	Creb3	ATF5,CREB3	0.45	2.1E-04	0.91	1.85E-03	-0.07	7.9E-01	-0.36	1.9E-01
1456021_at	Atf6	ATF6	0.26	4.5E-02	-0.46	2.56E-01	0.63	9.2E-03	0.72	2.2E-03
1449582_at	Cdx1	CDX1,2,4	0.06	6.4E-01	-0.25	5.58E-01	-0.32	2.3E-01	0.22	4.3E-01
1422074_at	Cdx2	CDX1,2,4	-0.01	9.3E-01	0.52	1.88E-01	-0.53	3.6E-02	-0.01	9.6E-01
1421552_at	Cdx4	CDX1,2,4	0.10	4.3E-01	-0.23	5.89E-01	0.15	5.7E-01	0.27	3.4E-01
1452901_at	Creb1	CREB1	0.59	4.5E-07	0.75	3.21E-02	-0.11	6.9E-01	-0.09	7.4E-01
1449042_at	Ctcf	CTCF	0.08	5.2E-01	0.25	5.49E-01	0.09	7.3E-01	0.30	2.7E-01
1418330_at	Ctcf	CTCF	0.21	1.0E-01	-0.12	7.83E-01	0.42	1.1E-01	-0.12	6.6E-01
1417878_at	E2f1	E2F	0.75	2.0E-12	-0.21	6.26E-01	0.58	1.9E-02	0.59	2.1E-02
1455790_at	E2f2	E2F	0.83	0.0E+00	0.80	1.83E-02	0.64	7.7E-03	0.59	2.0E-02
1434564_at	E2f3	E2F	0.20	1.3E-01	-0.23	5.89E-01	0.79	2.6E-04	0.59	2.0E-02
1451480_at	E2f4	E2F	-0.39	1.6E-03	0.18	6.70E-01	-0.88	8.7E-06	-0.16	5.7E-01
1447625_at	E2f5	E2F	0.23	6.7E-02	-0.50	2.09E-01	-0.56	2.6E-02	-0.21	4.5E-01
1448835_at	E2f6	E2F	0.15	2.4E-01	0.93	7.61E-04	0.49	5.2E-02	0.65	9.1E-03
1437187_at	E2f7	E2F	0.48	8.9E-05	0.45	2.66E-01	-0.06	8.3E-01	-0.53	4.0E-02
1436186_at	E2f8	E2F	0.78	1.0E-13	0.43	2.93E-01	0.55	2.8E-02	0.62	1.5E-02
1439319_at	Elf1	ELF1,2,4	0.18	1.7E-01	-0.40	3.26E-01	0.34	2.0E-01	0.23	4.0E-01
1428045_a_at	Elf2	ELF1,2,4	0.58	8.6E-07	0.57	1.39E-01	0.30	2.6E-01	0.33	2.2E-01
1421337_at	Elf4	ELF1,2,4	-0.14	2.7E-01	-0.21	6.21E-01	-0.37	1.6E-01	-0.42	1.2E-01
1446390_at	Elk1	ELK1,4,GABP{A,B1}	-0.02	8.7E-01	0.43	2.91E-01	-0.56	2.5E-02	-0.18	5.2E-01
1422233_at	Elk4	ELK1,4,GABP{A,B1}	-0.19	1.4E-01	-0.40	3.24E-01	-0.46	7.1E-02	-0.20	4.8E-01
1450665_at	Gabpa	ELK1,4,GABP{A,B1}	0.58	1.0E-06	0.03	9.38E-01	0.15	5.7E-01	0.05	8.7E-01
1436232_a_at	Gabpb1	ELK1,4,GABP{A,B1}	-0.13	3.1E-01	-0.53	1.76E-01	0.50	5.1E-02	0.17	5.5E-01
1460591_at	Esr1	ESR1	0.40	1.4E-03	0.87	4.48E-03	0.51	4.5E-02	0.66	7.9E-03
1425886_at	Fev	FEV	0.13	3.3E-01	-0.24	5.60E-01	0.35	1.9E-01	-0.16	5.7E-01
1423100_at	Fos	FOS,FOS{B,L1},JUN{B,D}	-0.14	2.9E-01	0.41	3.08E-01	0.55	2.9E-02	0.92	1.6E-06
1422134_at	Fosb	FOS,FOS{B,L1},JUN{B,D}	-0.08	5.5E-01	0.05	9.14E-01	0.29	2.7E-01	-0.03	9.2E-01
1417487_at	Fosl1	FOS,FOS{B,L1},JUN{B,D}	0.08	5.5E-01	-0.39	3.34E-01	0.74	9.3E-04	0.38	1.7E-01
1422931_at	Fosl2	FOSL2	-0.16	2.1E-01	-0.45	2.65E-01	0.63	8.8E-03	-0.34	2.1E-01
1434939_at	Foxf1	FOX{F1,F2,J1}	-0.57	1.2E-06	0.19	6.52E-01	-0.60	1.4E-02	-0.10	7.3E-01
1447562_at	Foxf2	FOX{F1,F2,J1}	0.38	2.2E-03	0.44	2.71E-01	0.15	5.8E-01	-0.05	8.7E-01
1425291_at	Foxj1	FOX{F1,F2,J1}	0.20	1.1E-01	-0.21	6.26E-01	-0.33	2.1E-01	-0.46	8.6E-02
1449458_at	Foxi1	FOX{I1,J2}	-0.32	1.0E-02	-0.05	9.16E-01	-0.15	5.8E-01	0.32	2.5E-01
1420374_at	Foxj2	FOX{I1,J2}	-0.11	4.0E-01	0.32	4.33E-01	0.23	3.9E-01	0.06	8.2E-01
1422210_at	Foxd3	FOXD3	-0.41	9.5E-04	-0.84	8.88E-03	0.26	3.2E-01	-0.35	2.1E-01
1423027_at	Foxl1	FOXL1	-0.03	8.1E-01	-0.68	6.33E-02	-0.47	6.7E-02	0.64	1.1E-02
1449232_at	Gata1	GATA1-3	-0.09	5.1E-01	-0.15	7.17E-01	0.20	4.5E-01	0.04	8.9E-01
1428816_a_at	Gata2	GATA1-3	0.02	8.9E-01	-0.15	7.15E-01	0.43	9.3E-02	0.36	1.8E-01
1448886_at	Gata3	GATA1-3	0.18	1.5E-01	-0.82	1.34E-02	0.05	8.6E-01	-0.71	2.8E-03
1425464_at	Gata6	GATA6	0.06	6.7E-01	-0.49	2.20E-01	-0.50	4.8E-02	-0.24	3.9E-01
1449058_at	Gli1	GLI1-3	0.11	3.9E-01	-0.19	6.56E-01	0.29	2.7E-01	0.62	1.3E-02
1446086_s_at	Gli2	GLI1-3	0.23	7.0E-02	-0.40	3.20E-01	-0.25	3.5E-01	-0.06	8.3E-01
1455154_at	Gli3	GLI1-3	0.18	1.5E-01	0.65	7.81E-02	0.01	9.8E-01	-0.01	9.6E-01
1450525_at	Gli3	GLI1-3	0.27	3.5E-02	0.17	6.92E-01	-0.54	3.0E-02	0.44	9.7E-02
1454631_at	Gtf2a1	GTF2A1-2	-0.60	2.5E-07	0.82	1.29E-02	-0.44	9.0E-02	-0.43	1.1E-01
1460367_at	Hbp1	HBP1,HMGB,SSRP1,UBTF	0.46	1.7E-04	-0.75	3.24E-02	0.26	3.4E-01	0.61	1.5E-02
1438307_at	Hmgb2	HBP1,HMGB,SSRP1,UBTF	0.13	3.2E-01	-0.65	7.81E-02	-0.41	1.1E-01	-0.51	5.3E-02
1416155_at	Hmgb3	HBP1,HMGB,SSRP1,UBTF	0.20	1.3E-01	-0.76	2.71E-02	-0.20	4.5E-01	-0.80	3.4E-04
1426788_a_at	Ssrp1	HBP1,HMGB,SSRP1,UBTF	0.09	4.9E-01	0.71	5.05E-02	-0.70	2.6E-03	-0.49	6.5E-02
1460304_a_at	Ubtf	HBP1.HMGB.SSRP1.UBTF	0.69	6.7E-10	-0.18	6.69E-01	-0.12	6.5E-01	0.17	5.5E-01
1434736_at	Hlf	HLF	-0.41	1.0E-03	0.10	8.20E-01	-0.68	4.0E-03	0.35	2.0E-01
1421234_at	Hnf1a	HNF1A	0.07	6.1E-01	0.45	2.63E-01	0.22	4.2E-01	-0.25	3.7E-01
1427000_at	Hnf4a	HNF4A,NR2F1,2	-0.02	9.0E-01	-0.43	2.86E-01	-0.35	1.8E-01	-0.64	1.1E-02
1418157_at	Nr2f1	HNF4A,NR2F1,2	-0.33	9.4E-03	-0.47	2.45E-01	-0.22	4.1E-01	0.40	1.4E-01
1416159_at	Nr2f2	HNF4A,NR2F1,2	0.45	2.3E-04	0.10	8.10E-01	-0.41	1.2E-01	0.69	4.1E-03
1427354_at	Hoxa4	HOX{A4,D4}	0.05	7.2E-01	0.16	7.12E-01	0.72	1.6E-03	0.20	4.8E-01
1450209_at	Hoxd4	HOX{A4,D4}	0.04	7.7E-01	0.40	3.29E-01	0.03	9.1E-01	0.11	7.0E-01
1448436_a_at	Irf1	IRF1,2,7	0.49	5.8E-05	0.10	8.17E-01	0.44	8.9E-02	0.87	2.4E-05
1418265_s_at	Irf2	IRF1,2,7	-0.20	1.2E-01	0.36	3.87E-01	-0.35	1.8E-01	-0.15	5.9E-01
1417244_a_at	Irf7	IRF1,2,7	0.71	1.0E-10	0.75	3.30E-02	0.61	1.2E-02	0.19	5.1E-01
1439846_at	Klf12	KLF12	-0.42	7.9E-04	0.57	1.41E-01	0.55	2.7E-02	0.04	9.0E-01
1417395_at	Klf4	KLF4	0.00	9.7E-01	0.75	3.36E-02	0.46	7.3E-02	-0.20	4.8E-01

Table S6: Pearson correlation coefficient (PCC) and associate *P*-values between motif activities and mRNA expression of cognate transcription factors in each data-sets - **part 1**. Part 2 in Table S7. Affx = probe-set ID from Affymetrix platform Mouse 430_2. GS = gene symbol. PCC = Pearson correlation coefficient.

Affx	GS	Motifs	Kinetic study PCC	Kinetic study P-value	β-catenin study PCC	β-catenin study P-value	CAR KO study PCC	CAR KO study P-value	Tumor Study PCC	Tumor Study P-value
1454734_at	Lef1	LEF1,TCF7,TCF7L1,2	-0.03	8.4E-01	0.30	4.73E-01	-0.25	3.5E-01	0.49	6.2E-02
1433471_at	Tcf7	LEF1,TCF7,TCF7L1,2	0.22	8.2E-02	0.46	2.52E-01	-0.26	3.3E-01	0.32	2.4E-01
1450117_at	Tcf7l1	LEF1,TCF7,TCF7L1,2	0.22	8.0E-02	-0.76	2.86E-02	0.25	3.5E-01	-0.43	1.1E-01
1426639_a_at	Tcf7l2	LEF1,TCF7,TCF7L1,2	0.32	1.2E-02	0.38	3.53E-01	0.45	8.3E-02	0.20	4.8E-01
1454086_a_at	Lmo2	LMO2	-0.01	9.7E-01	0.22	5.95E-01	-0.60	1.5E-02	-0.25	3.6E-01
1429170_a_at	Mtf1	MTF1	-0.55	3.4E-06	0.54	1.70E-01	-0.34	2.0E-01	-0.09	7.4E-01
1421317_x_at	Myb	MYB	-0.20	1.2E-01	-0.33	4.19E-01	-0.07	8.1E-01	-0.24	4.0E-01
1452001_at	Nfe2	NFE2	-0.27	3.5E-02	-0.71	4.74E-02	0.76	6.0E-04	0.22	4.3E-01
1457117_at	Nfe2l2	NFE2L2	-0.35	4.8E-03	-0.35	3.97E-01	-0.21	4.3E-01	0.22	4.2E-01
1418932_at	Nfil3	NFIL3	-0.07	5.7E-01	-0.31	4.49E-01	-0.55	2.7E-02	-0.10	7.3E-01
1427705_a_at	Nfkb1	NFKB1,REL,RELA	0.24	5.8E-02	-0.83	1.02E-02	0.48	5.8E-02	0.28	3.1E-01
1420710_at	Rel	NFKB1,REL,RELA	-0.44	3.1E-04	0.07	8.76E-01	0.18	5.2E-01	0.02	9.3E-01
1419536_a_at	Rela	NFKB1,REL,RELA	0.14	2.9E-01	0.55	1.62E-01	0.19	4.9E-01	0.13	6.3E-01
1427808_at	Nfya	NFY{A,B,C}	-0.08	5.2E-01	0.24	5.73E-01	-0.35	1.9E-01	-0.47	7.8E-02
1419266_at	Nfyb	NFY{A,B,C}	0.32	1.2E-02	0.58	1.36E-01	0.70	2.6E-03	0.73	2.1E-03
1448963_at	Nfyc	NFY{A,B,C}	0.35	4.9E-03	0.36	3.87E-01	0.17	5.3E-01	-0.28	3.1E-01
1421112_at	Nkx2-2	NKX2-2,8	-0.31	1.3E-02	0.35	3.96E-01	-0.09	7.3E-01	0.09	7.4E-01
1422284_at	Nkx2-9	NKX2-2,8	-0.46	1.9E-04	-0.10	8.21E-01	-0.17	5.3E-01	-0.19	4.9E-01
1421464_at	Nkx3-2	NKX3-2	0.21	9.5E-02	0.18	6.78E-01	-0.11	6.9E-01	-0.01	9.8E-01
1419105_at	Nr1h4	NR1H4	-0.16	2.0E-01	-0.19	6.56E-01	0.38	1.5E-01	0.66	7.0E-03
1421730_at	Nr5a1	NR5A1,2	0.26	4.4E-02	-0.20	6.29E-01	0.79	3.1E-04	0.37	1.7E-01
1449707_at	Nr5a2	NR5A1,2	0.07	5.8E-01	-0.29	4.79E-01	0.21	4.4E-01	-0.41	1.3E-01
1421515_at	Nr6a1	NR6A1	0.24	5.9E-02	0.37	3.69E-01	0.17	5.3E-01	0.38	1.6E-01
1424787_a_at	Nrf1	NRF1	0.55	4.2E-06	-0.36	3.80E-01	0.33	2.0E-01	-0.08	7.9E-01
1460044_at	Onecut2	ONECUT1,2	-0.27	3.3E-02	0.77	2.50E-02	0.41	1.2E-01	-0.29	2.9E-01
1428647_at	Pbx1	PBX1	0.29	2.2E-02	-0.37	3.63E-01	0.47	6.8E-02	0.10	7.4E-01
1416967_at	Sox2	POU5F1,SOX2{dimer}	0.45	2.7E-04	0.08	8.46E-01	0.20	4.6E-01	-0.23	4.1E-01
1452844_at	Pou6f1	POU6F1	0.51	2.6E-05	-0.68	6.22E-02	0.21	4.3E-01	0.00	1.0E+00
1420425_at	Prdm1	PRDM1	0.00	9.9E-01	-0.09	8.36E-01	0.36	1.7E-01	0.48	7.1E-02
1428227_at	Rest	REST	-0.64	2.3E-08	-0.62	9.92E-02	-0.54	3.1E-02	-0.06	8.3E-01
1436059_at	Rfx1	RFX1-5,RFXANK,RFXAP	-0.10	4.4E-01	0.57	1.43E-01	-0.36	1.7E-01	-0.43	1.1E-01
1442578_at	Rfx2	RFX1-5,RFXANK,RFXAP	0.23	7.3E-02	0.62	1.03E-01	0.55	2.6E-02	0.09	7.5E-01
1425413_at	Rfx3	RFX1-5,RFXANK,RFXAP	0.27	3.5E-02	-0.77	2.68E-02	0.49	5.4E-02	0.19	5.1E-01
1436931_at	Rfx4	RFX1-5,RFXANK,RFXAP	0.00	9.8E-01	0.78	2.20E-02	-0.59	1.6E-02	-0.32	2.5E-01
1425670_at	Rfxank	RFX1-5,RFXANK,RFXAP	0.44	3.9E-04	-0.11	7.94E-01	0.41	1.2E-01	0.23	4.0E-01
1455303_at	Rfxap	RFX1-5,RFXANK,RFXAP	0.53	1.1E-05	-0.08	8.59E-01	-0.34	2.0E-01	-0.10	7.1E-01
1440878_at	Runx1	RUNX1-3	0.05	6.8E-01	0.64	8.89E-02	0.15	5.7E-01	0.43	1.1E-01
1425389_a_at	Runx2	RUNX1-3	0.28	2.8E-02	-0.63	9.54E-02	0.29	2.8E-01	0.35	2.0E-01
1421467_at	Runx3	RUNX1-3	-0.06	6.7E-01	0.59	1.22E-01	0.65	5.9E-03	0.31	2.7E-01
1454773_at	Rxra	RXR{A,B,G}	0.27	3.7E-02	0.88	3.55E-03	-0.17	5.4E-01	0.43	1.1E-01
1416990_at	Rxrb	RXR{A,B,G}	-0.26	4.3E-02	0.09	8.27E-01	-0.04	8.9E-01	0.14	6.3E-01
1418782_at	Rxrg	RXR{A,B,G}	0.19	1.4E-01	0.24	5.68E-01	0.59	1.5E-02	-0.36	1.8E-01
1451689_a_at	Sox10	SOX{8,9,10}	0.02	8.5E-01	-0.49	2.21E-01	-0.66	5.3E-03	-0.59	2.1E-02
1435438_at	Sox8	SOX{8,9,10}	-0.06	6.7E-01	-0.23	5.82E-01	0.17	5.2E-01	0.40	1.4E-01
1451538_at	Sox9	SOX{8,9,10}	-0.24	5.5E-02	0.74	3.66E-02	-0.64	7.7E-03	-0.19	5.0E-01
1418747_at	Sfpi1	SPI1	-0.06	6.4E-01	0.08	8.59E-01	0.51	4.5E-02	0.25	3.6E-01
1418256_at	Srf	SRF	0.26	4.4E-02	0.22	6.08E-01	-0.02	9.5E-01	0.80	3.6E-04
1426470_at	Tbp	TBP	-0.07	6.0E-01	0.07	8.74E-01	-0.46	7.1E-02	-0.61	1.5E-02
1429556_at	Tead1	TEAD1	-0.02	9.0E-01	0.72	4.59E-02	0.40	1.2E-01	-0.13	6.5E-01
1436392_s_at	Tfap2c	TFAP{A,C}	0.07	6.1E-01	-0.70	5.47E-02	0.26	3.3E-01	0.45	9.2E-02
1426048_s_at	Tfap2a	TFAP2{A,C}	0.44	3.3E-04	0.36	3.75E-01	0.27	3.1E-01	0.47	7.4E-02
1435670_at	Tfap2b	TFAP2B	-0.36	4.6E-03	-0.02	9.67E-01	-0.61	1.1E-02	-0.05	8.5E-01
1418167_at	Tfap4	TFAP4	0.15	2.6E-01	-0.75	3.24E-02	-0.13	6.2E-01	-0.18	5.3E-01
1418159_at	Tfcp2	TFCP2	-0.30	1.7E-02	-0.46	2.48E-01	-0.22	4.0E-01	-0.21	4.5E-01
1455273_at	Zbtb6	ZBTB6	0.69	8.0E-10	0.52	1.83E-01	-0.39	1.3E-01	-0.01	9.7E-01
1420865_at	Zbtb14	ZFP161	0.40	1.2E-03	-0.52	1.86E-01	-0.52	3.8E-02	0.30	2.8E-01
1422599_s_at	Zfp143	ZNF143	0.41	8.4E-04	0.65	7.98E-02	0.74	1.1E-03	-0.14	6.1E-01
1436217_at	Zfp148	ZNF148	-0.73	1.7E-11	-0.46	2.56E-01	-0.28	3.0E-01	-0.72	2.3E-03
1438047_at	Zfp384	ZNF384	0.08	5.2E-01	-0.43	2.85E-01	0.08	7.6E-01	-0.19	4.9E-01

Table S7: Pearson correlation coefficient (PCC) and associate P-values between motif activities and mRNA expression of cognate transcription factors in each data-sets - **part 2**. Affx = probe-set ID from Affymetrix platform Mouse 430_2. GS = gene symbol. PCC = Pearson correlation coefficient.

References

[1] Lempiainen, H., et al. (2013 Feb) Identification of dlk1-dio3 imprinted gene cluster noncoding rnas as novel candidate biomarkers for liver tumor promotion. *Toxicol Sci*, **131**, 375–386.

[2] Tan, X., Behari, J., Cieply, B., Michalopoulos, G. K., and Monga, S. P. S. (2006 Nov) Conditional deletion of beta-catenin reveals its role in liver growth and regeneration. *Gastroenterology*, **131**, 1561–1572.

[3] Phillips, J. M., Burgoon, L. D., and Goodman, J. I. (2009 Aug) The constitutive active/androstane receptor facilitates unique phenobarbital-induced expression changes of genes involved in key pathways in precancerous liver and liver tumors. *Toxicol Sci*, **110**, 319–333.

[4] R Core Team (2013) *R: A Language and Environment for Statistical Computing*. R Foundation for Statistical Computing, Vienna Austria.

[5] Kauffmann, A., Gentleman, R., and Huber, W. (2009 Feb 1) arrayqualitymetrics–a bioconductor package for quality assessment of microarray data. *Bioinformatics*, **25**, 415–416.

[6] Septer, S., Edwards, G., Gunewardena, S., Wolfe, A., Li, H., Daniel, J., and Apte, U. (2012) Yes-associated protein is involved in proliferation and differentiation during postnatal liver development. *Am J Physiol Gastrointest Liver Physiol*, **302**, G493–503.

[7] Hart, S. N., Cui, Y., Klaassen, C. D., and Zhong, X.-b. (2009) Three patterns of cytochrome p450 gene expression during liver maturation in mice. *Drug Metab Dispos*, **37**, 116–21.

[8] Helin, K. (1998) Regulation of cell proliferation by the {E2F} transcription factors. *Current Opinion in Genetics & Development*, **8**, 28 – 35.

[9] Rodriguez, J. L., et al. (2007) Transcription of the mat2a gene, coding for methionine adenosyltransferase, is up-regulated by e2f and sp1 at a chromatin level during proliferation of liver cells. *Int J Biochem Cell Biol*, **39**, 842–850.

[10] Wu, L., et al. (2001 Nov 22) The e2f1-3 transcription factors are essential for cellular proliferation. *Nature*, **414**, 457–462.

[11] Gille, H., Sharrocks, A. D., and Shaw, P. E. (1992 Jul 30) Phosphorylation of transcription factor p62tcf by map kinase stimulates ternary complex formation at c-fos promoter. *Nature*, **358**, 414–417.

[12] Baena, E., Gandarillas, A., Vallespinos, M., Zanet, J., Bachs, O., Redondo, C., Fabregat, I., Martinez-A, C., and de Alboran, I. M. (2005 May 17) c-myc regulates cell size and ploidy but is not essential for postnatal proliferation in liver. *Proc Natl Acad Sci U S A*, **102**, 7286–7291.

[13] Goldsworthy, T. L., Goldsworthy, S. M., Sprankle, C. S., and Butterworth, B. E. (1994 May) Expression of myc, fos and ha-ras associated with chemically induced cell proliferation in the rat liver. *Cell Prolif*, **27**, 269–278.

[14] Cui, Y. J., Yeager, R. L., Zhong, X.-B., and Klaassen, C. D. (2009) Ontogenic expression of hepatic ahr mrna is associated with histone h3k4 di-methylation during mouse liver development. *Toxicol Lett*, **189**, 184–90.

[15] Chesire, D. R., Dunn, T. A., Ewing, C. M., Luo, J., and Isaacs, W. B. (2004 Apr 1) Identification of aryl hydrocarbon receptor as a putative wnt/beta-catenin pathway target gene in prostate cancer cells. *Cancer Res*, **64**, 2523–2533.

[16] Maher, J. M., Cheng, X., Slitt, A. L., Dieter, M. Z., and Klaassen, C. D. (2005) Induction of the multidrug resistance-associated protein family of transporters by chemical activators of receptor-mediated pathways in mouse liver. *Drug Metab Dispos*, **33**, 956–62.

[17] Alnouti, Y. and Klaassen, C. D. (2008) Tissue distribution, ontogeny, and regulation of aldehyde dehydrogenase (aldh) enzymes mrna by prototypical microsomal enzyme inducers in mice. *Toxicol Sci*, **101**, 51–64.

[18] Lahvis, G. P. and Bradfield, C. A. (1998) Ahr null alleles: distinctive or different? *Biochem Pharmacol*, **56**, 781–787.

[19] Huang, D. W., Sherman, B. T., and Lempicki, R. A. (2009 Jan) Bioinformatics enrichment tools: paths toward the comprehensive functional analysis of large gene lists. *Nucleic Acids Res*, **37**, 1–13.

[20] Huang, D. W., Sherman, B. T., and Lempicki, R. A. (2009) Systematic and integrative analysis of large gene lists using david bioinformatics resources. *Nat Protoc*, **4**, 44–57.
